# Supplementary material for: Proteogenomics Reveals Orthologous Alternatively Spliced Proteoforms in the Same Human and Mouse Brain Regions with Differential Abundance in an Alzheimer’s Disease Mouse Model
Source: Cells. 2021 Jun 23;10(7):1583. doi: 10.3390/cells10071583 (PMC8303486; doi:10.3390/cells10071583)
Supplement: Supplementary file 1 [file cells-10-01583-s001.zip › Figure S1 - Evaluation of total RNA Integrity.pdf]

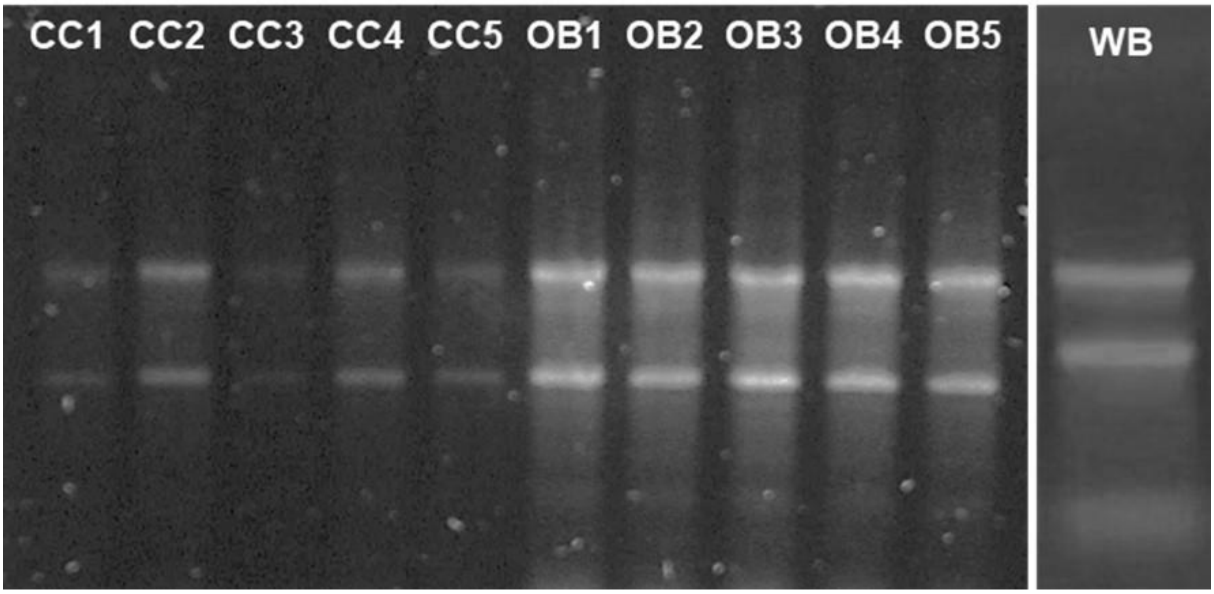

Figure S1. Evaluation of total RNA Integrity. Total RNA from corpus callosum (CC, n=5), olfactory bulb (OB, n=5) and whole brain (WB, n=1) were extracted using the TRIzol reagent (Sigma). Different sample amounts of total RNA from CC (CC 1: 200 ng; CC 2: 400 ng; CC3: 180 ng; CC 4: 300 ng; CC 5: 300 ng), 400 ng of total RNA from each OB (OB1 to 5) and from 400ng of whole brain (WB) RNA were analyzed by non- denaturing 1% agarose gel electrophoresis in 1X tris-acetate-EDTA (TAE) buffer stained with SYBR® Safe (Invitrogen). After the electrophoresis, gel was observed and photographed under ultraviolet light (UV).
