# Supplementary material for: Proteogenomics Reveals Orthologous Alternatively Spliced Proteoforms in the Same Human and Mouse Brain Regions with Differential Abundance in an Alzheimer’s Disease Mouse Model
Source: Cells. 2021 Jun 23;10(7):1583. doi: 10.3390/cells10071583 (PMC8303486; doi:10.3390/cells10071583)
Supplement: Supplementary file 1 [file cells-10-01583-s001.zip › Figure S2 - Evaluation of the quality of the synthesized cDNA.pdf]

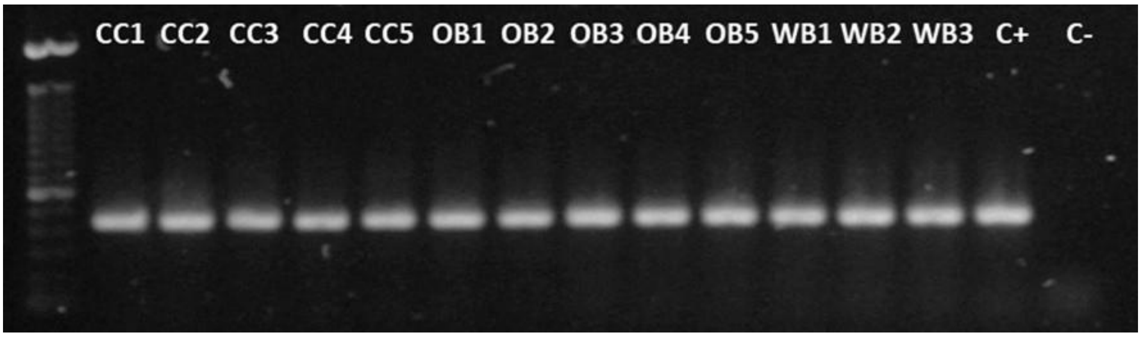

Figure S2. Evaluation of the quality of the synthesized cDNA. For cDNA synthesis, 1 µg of the total RNA of each sample was submitted to reverse transcription (RT). The quality of the synthesized cDNA was evaluated by the PCR amplification of the glyceraldehyde 3-phosphate dehydrogenase (*Gapdh*) gene. The amplification product was assessed by 1.2% agarose gel electrophoresis in 1X tris-acetate-EDTA (TAE) buffer stained with SYBR® Safe (Invitrogen) and observed under UV light. CC: Corpus callosum samples; OB: olfactory bulb samples; C+: SaI/N cells cDNA as positive control; C-: No template as negative control.
