# Supplementary material for: Proteogenomics Reveals Orthologous Alternatively Spliced Proteoforms in the Same Human and Mouse Brain Regions with Differential Abundance in an Alzheimer’s Disease Mouse Model
Source: Cells. 2021 Jun 23;10(7):1583. doi: 10.3390/cells10071583 (PMC8303486; doi:10.3390/cells10071583)
Supplement: Supplementary file 1 [file cells-10-01583-s001.zip › Figure S3 - Dissociation curves obtained after optimization of RT-qPCR for amplification of Prkcb, Stxbp1, Cadm1, Hnrnpk, Pkm, Crmpd1 and Hprt1 genes from .pdf]

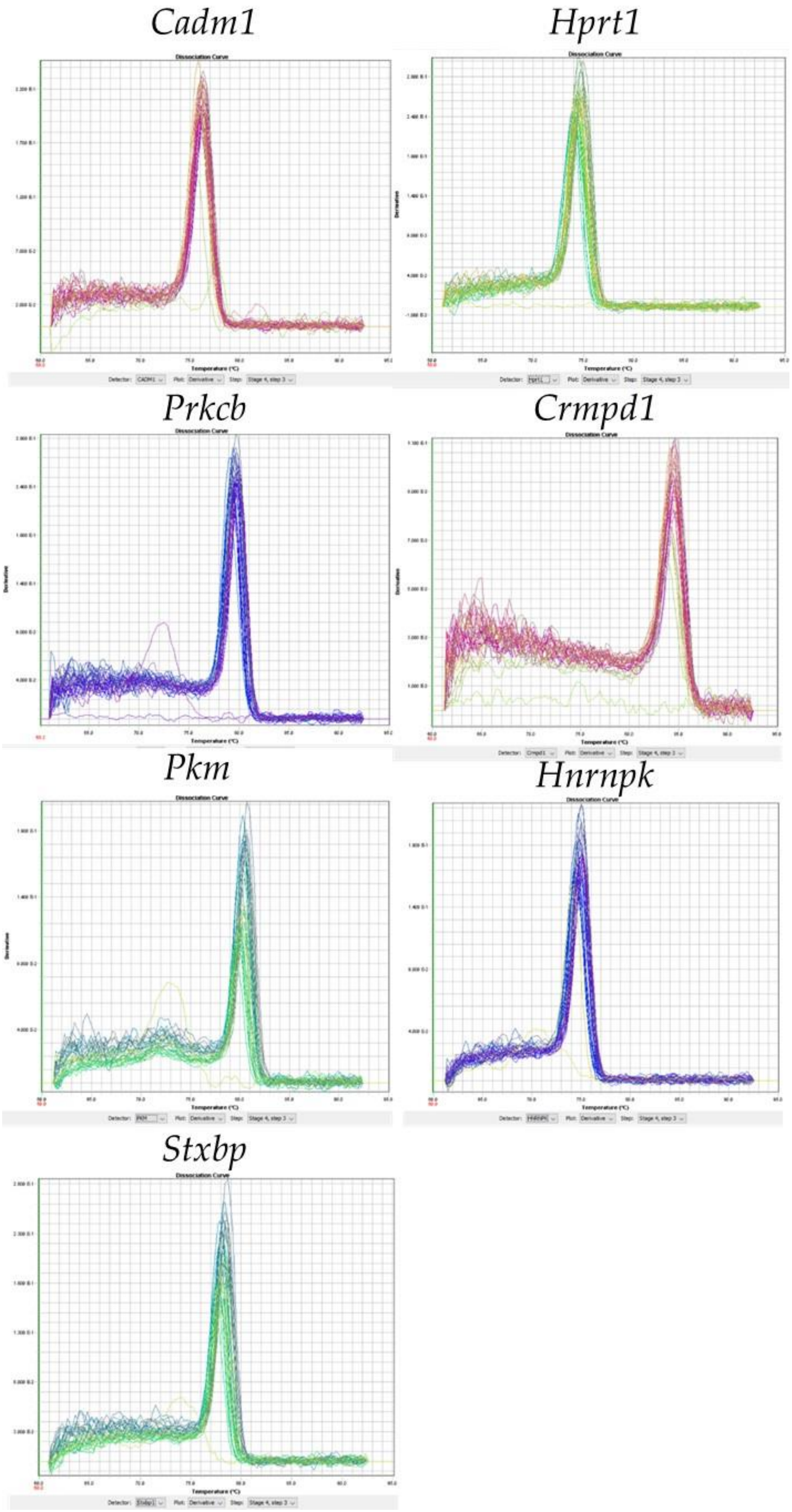

Figure S3. Dissociation curves obtained after optimization of RT-qPCR for amplification of *Prkcb*, *Stxbp1*, *Cadm1*, *Hnrnpk*, *Pkm*, *Crmpd1* and *Hprt1* genes from brain tissues. cDNA (2 µL of a 1: 5 dilution) from brain tissues was used as template in RT-qPCR using the SYBR green Master Mix Detection System. All reactions were run in 7900HT Fast Real-Time PCR System (Applied Biosystems) and the dissociation curves were obtained using the following thermocycler parameters: 95 ° C 15 ', 60 ° C 15' and 95 ° C 15 '.
