# Supplementary material for: Proteogenomics Reveals Orthologous Alternatively Spliced Proteoforms in the Same Human and Mouse Brain Regions with Differential Abundance in an Alzheimer’s Disease Mouse Model
Source: Cells. 2021 Jun 23;10(7):1583. doi: 10.3390/cells10071583 (PMC8303486; doi:10.3390/cells10071583)
Supplement: Supplementary file 1 [file cells-10-01583-s001.zip › Figure S4 - Schematic representation of the spliced transcripts of the genes Cadm1, Stxbp1, Pkm, Prkcb, Hnrnpk, Crmp1 and the annealing positions of their .pdf]

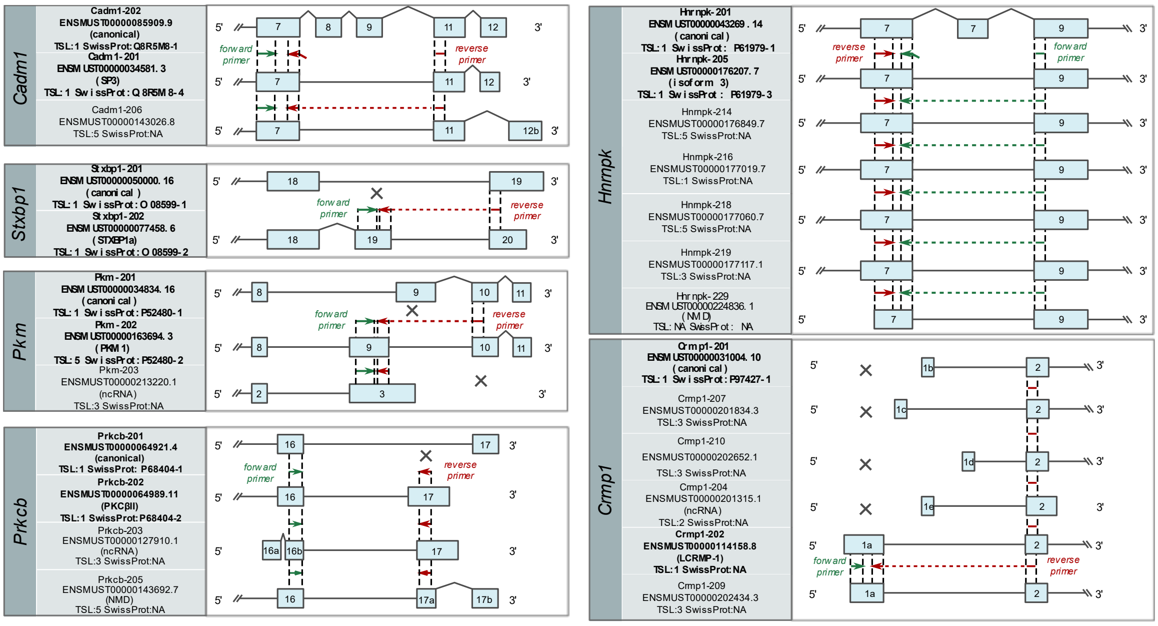

Figure S4. Schematic representation of the spliced transcripts of the genes: *Cadm1*; *Stxbp1*; *Pkm*; *Prkcb*; *Hnrnpk*; *Crmp1* and the annealing positions of their primers among exons. Exons are represented by boxes, and introns are represented by lines. Each primer is represented by green (foward) and red (reverse) arrows. The dashed lines of the arrows represet primers spanning more than one exon. The two slashes in the intronic region indicate a fragment of mRNA structure not represented in this scheme.
