# Supplementary material for: Proteogenomics Reveals Orthologous Alternatively Spliced Proteoforms in the Same Human and Mouse Brain Regions with Differential Abundance in an Alzheimer’s Disease Mouse Model
Source: Cells. 2021 Jun 23;10(7):1583. doi: 10.3390/cells10071583 (PMC8303486; doi:10.3390/cells10071583)
Supplement: Supplementary file 1 [file cells-10-01583-s001.zip › Figure S5 - MSMS spectra of the identical peptides.pdf]

Human Olfactory bulb
LFEELVR

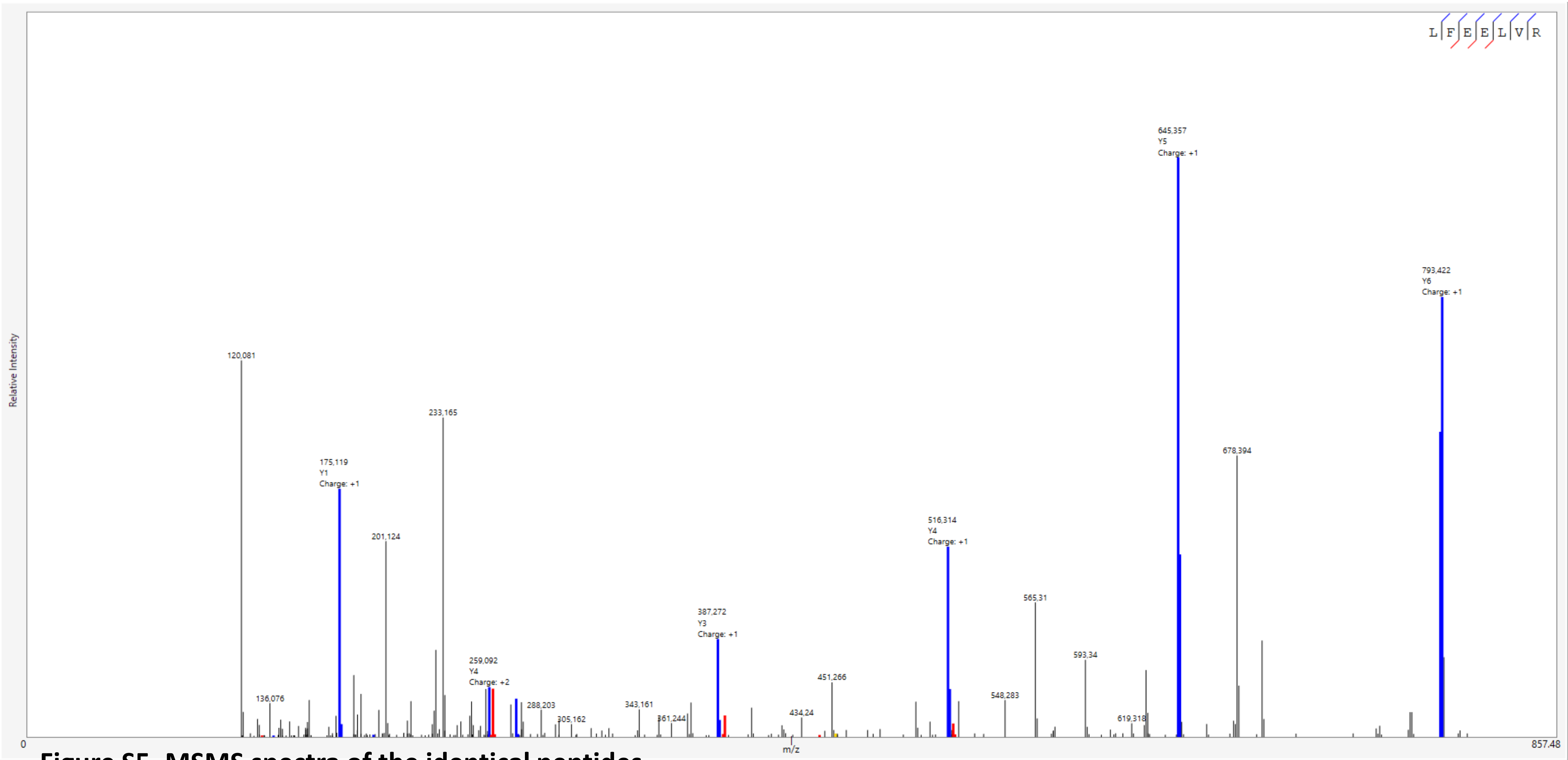

Figure S5. MSMS spectra of the identical peptides.

Human Olfactory bulb
ASSHSTDLMEAMAMGSVEASYK

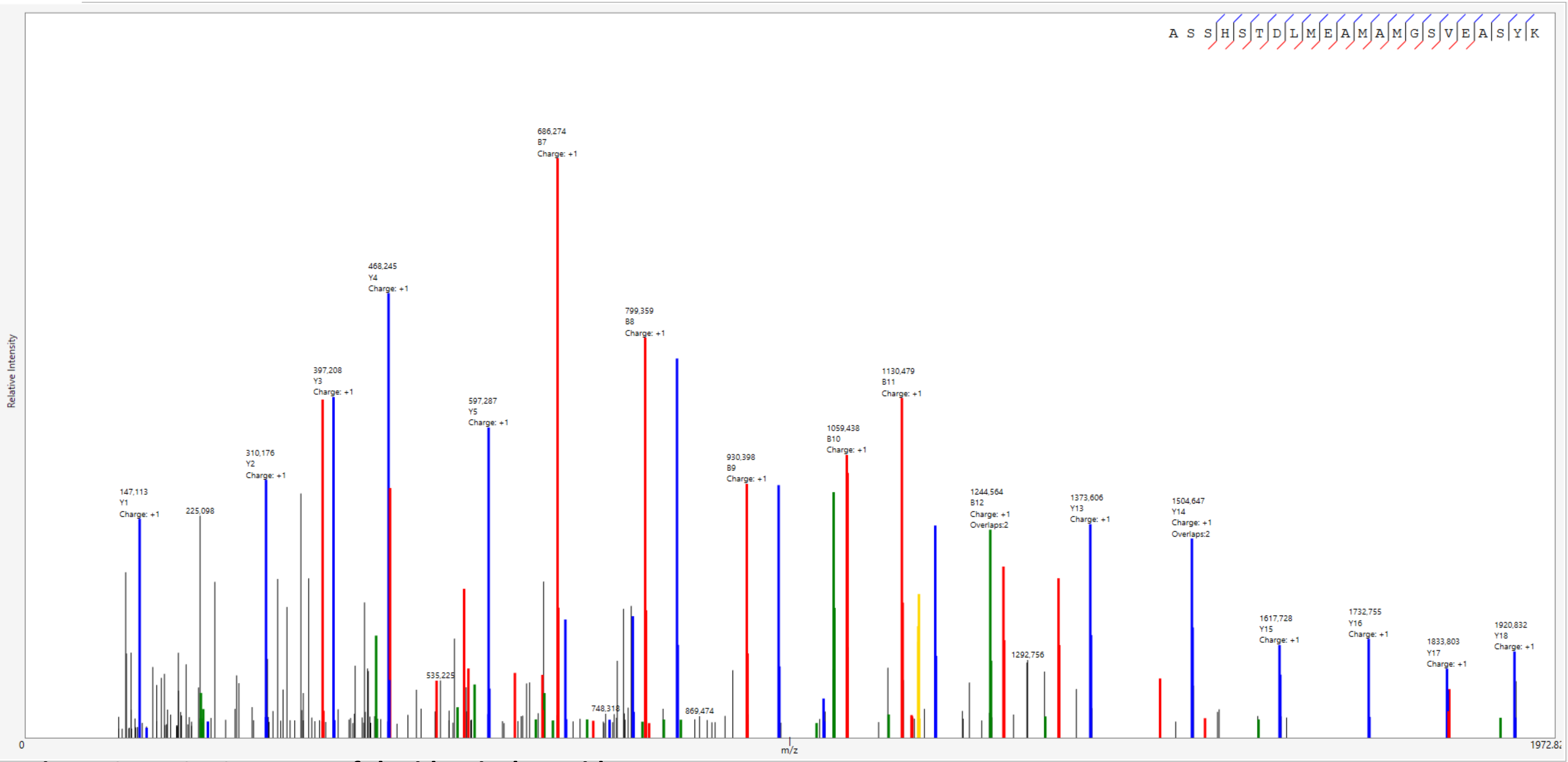

Figure S5. MSMS spectra of the identical peptides.

Human Corpus Callosum
CLAAALIVLTESGR

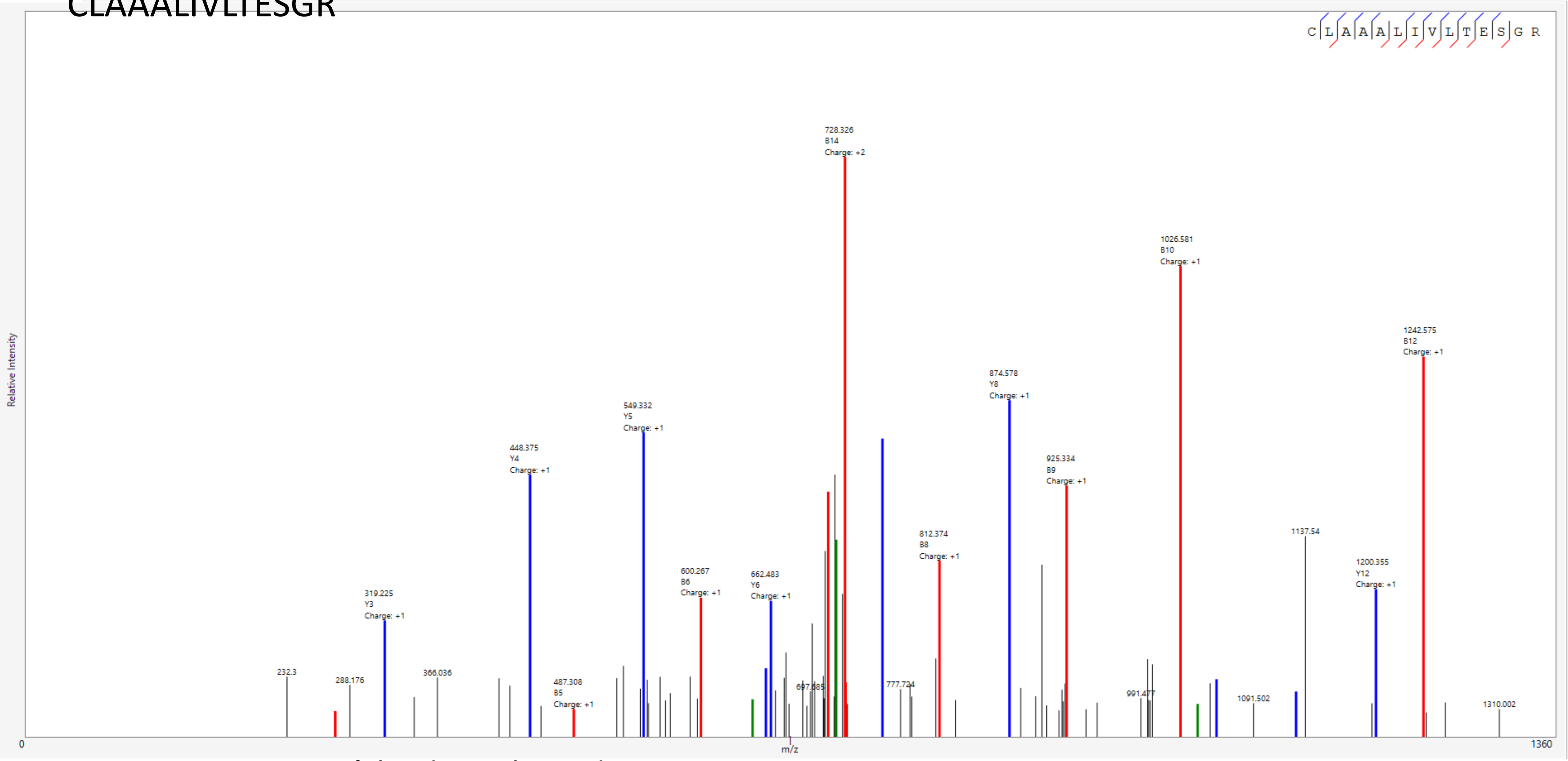

Figure S5. MSMS spectra of the identical peptides.

Human Olfactory bulb
CLAAAALIVLTESGR

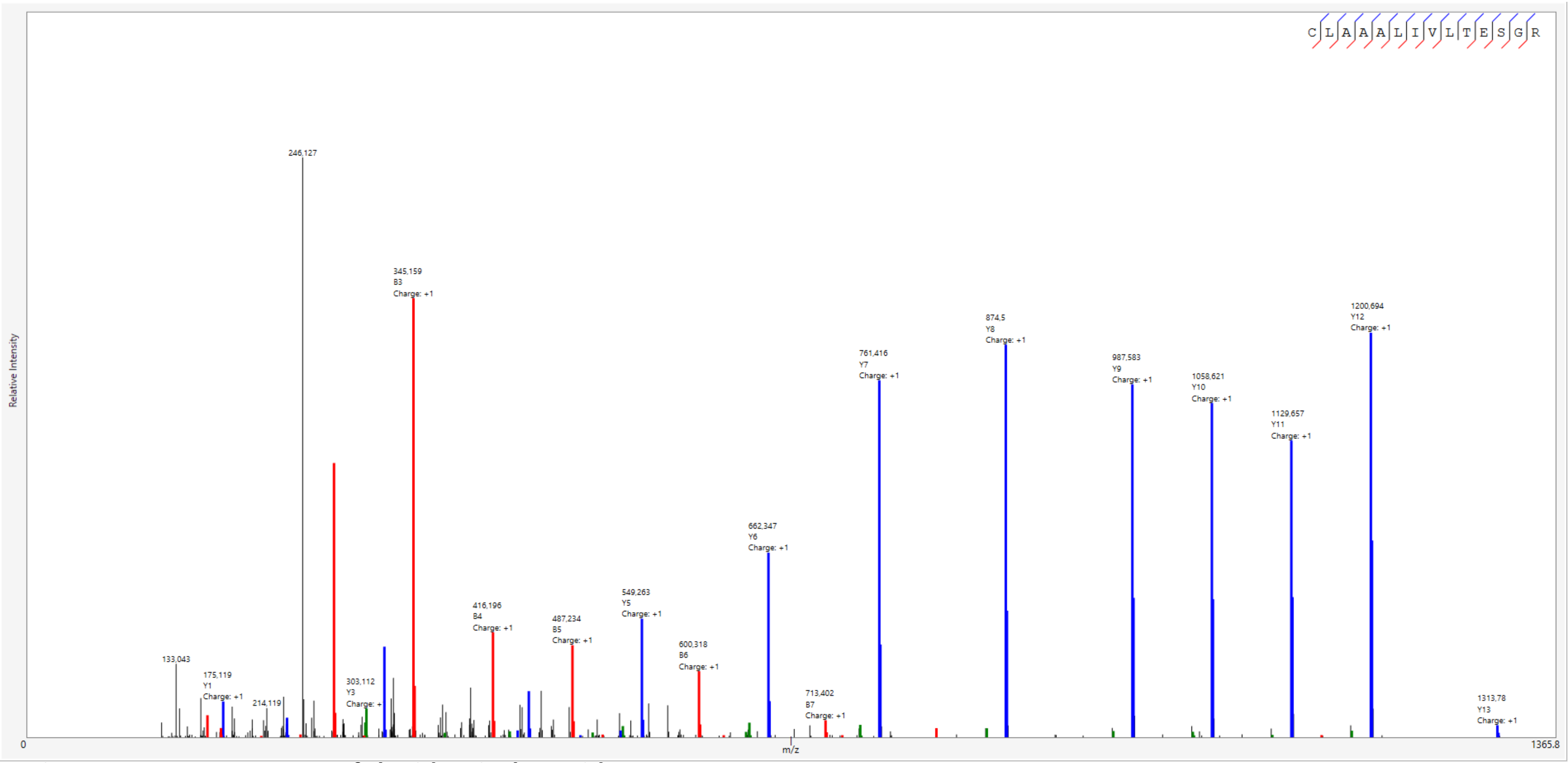

Figure S5. MSMS spectra of the identical peptides.

Human Olfactory bulb
EAEAAMFHR

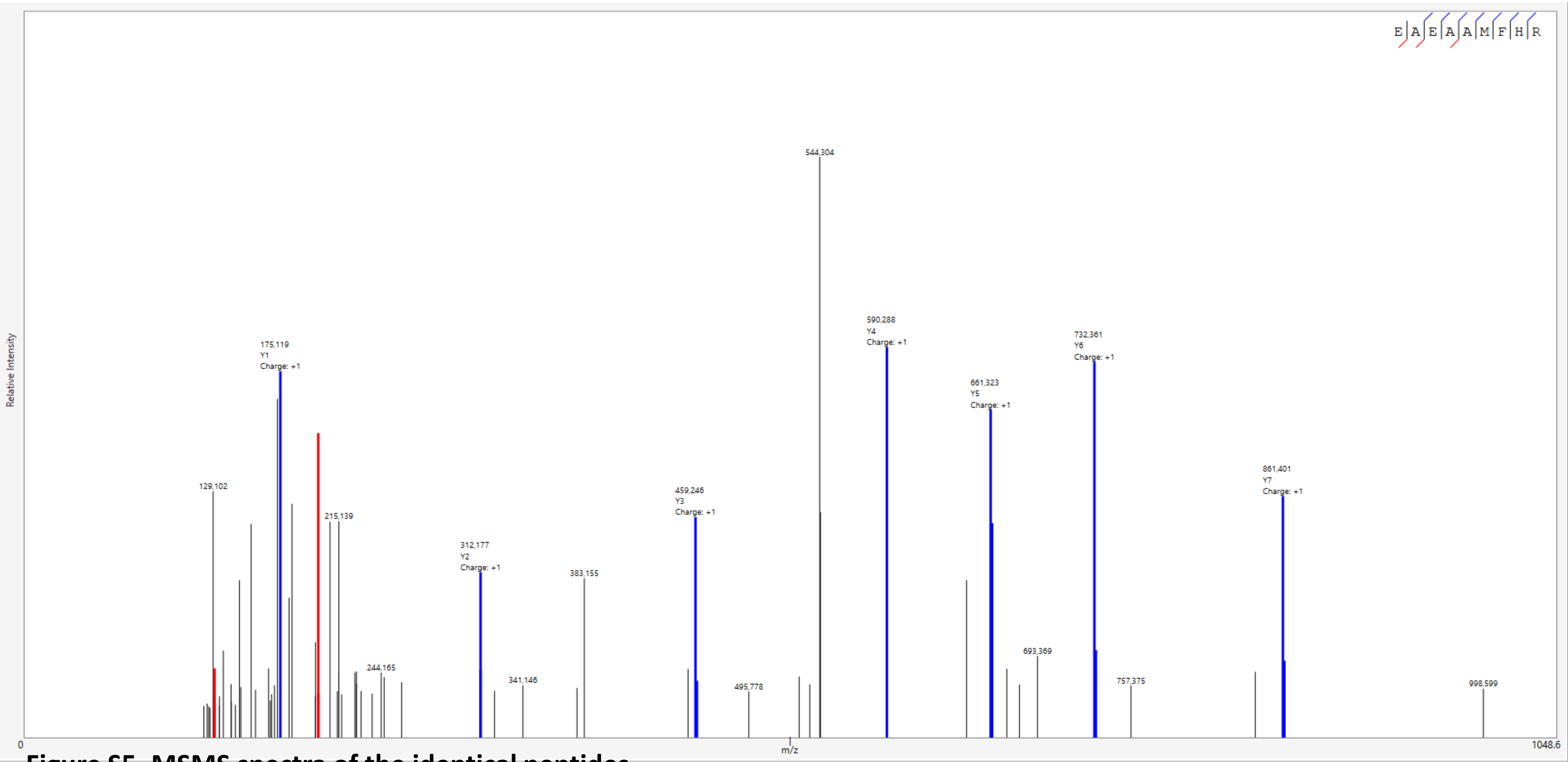

Figure S5. MSMS spectra of the identical peptides.

Human Olfactory bulb
IIPTLEEYQHYK

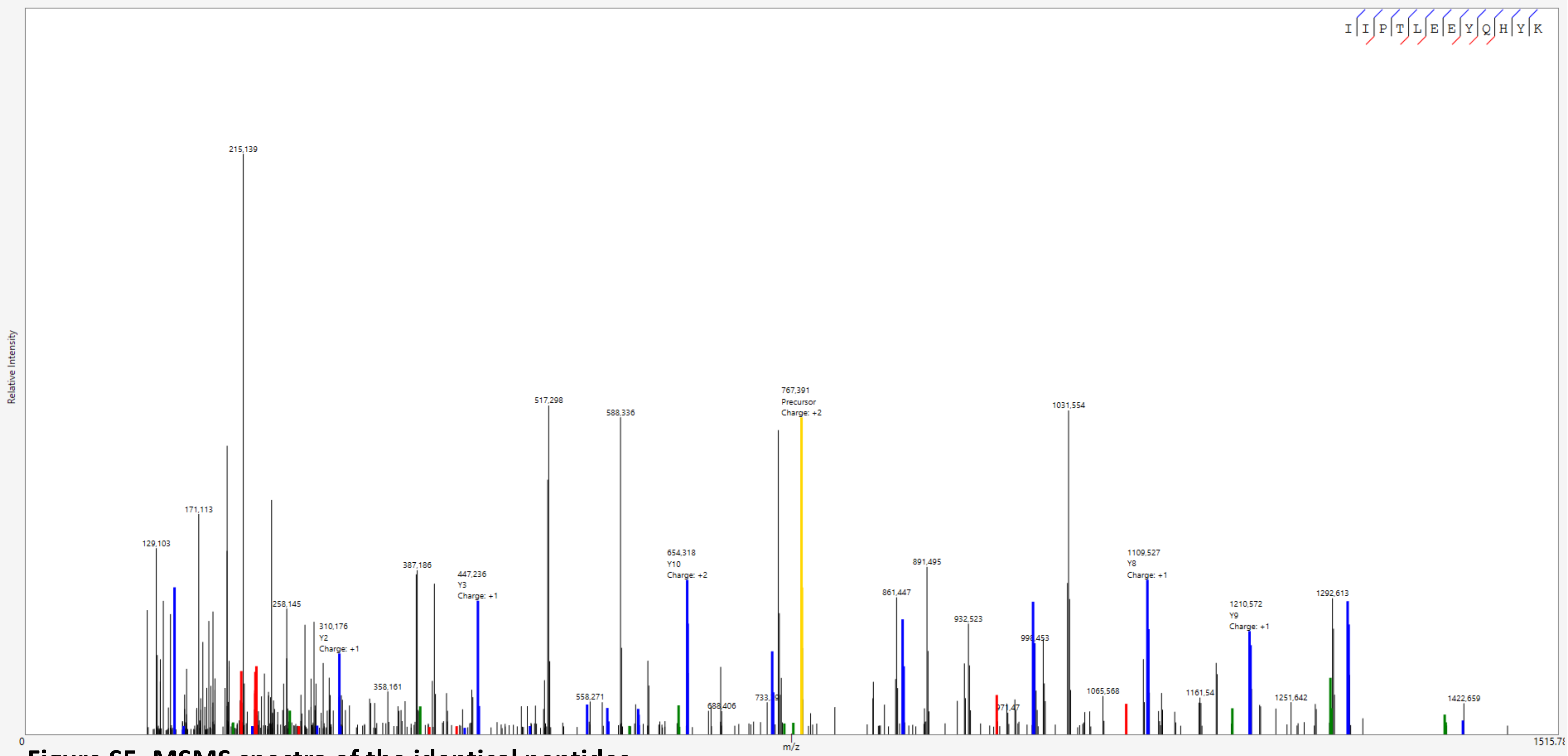

Figure S5. MSMS spectra of the identical peptides.

Human Olfactory bulb
NIDQSEFEGFSFVNSEFLKPEVK

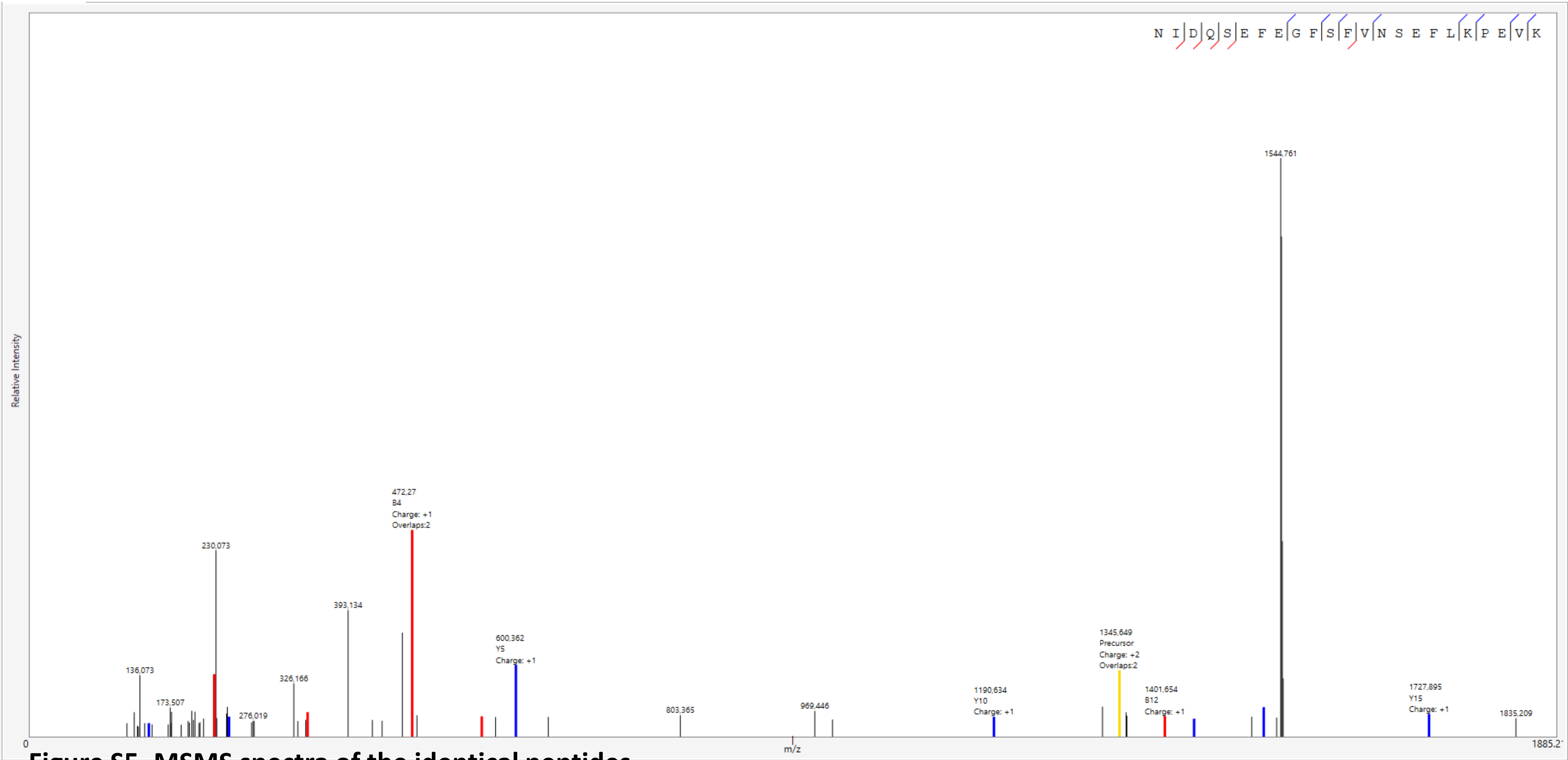

Figure S5. MSMS spectra of the identical peptides.

Human Olfactory bulb
WEVLIGSTHILTPTK

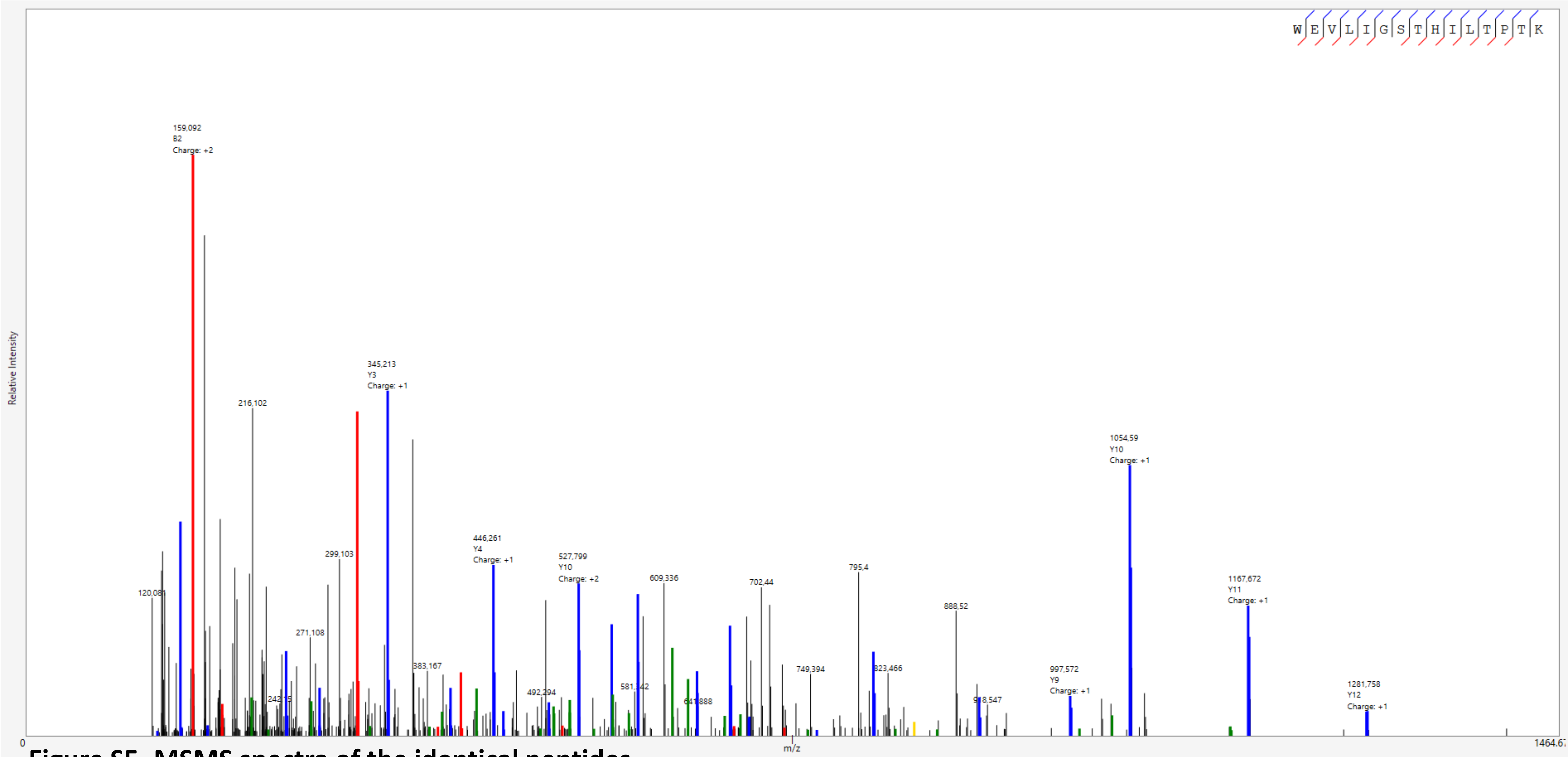

Figure S5. MSMS spectra of the identical peptides.

Human Olfactory bulb
YGGMFAAVEGAYENK

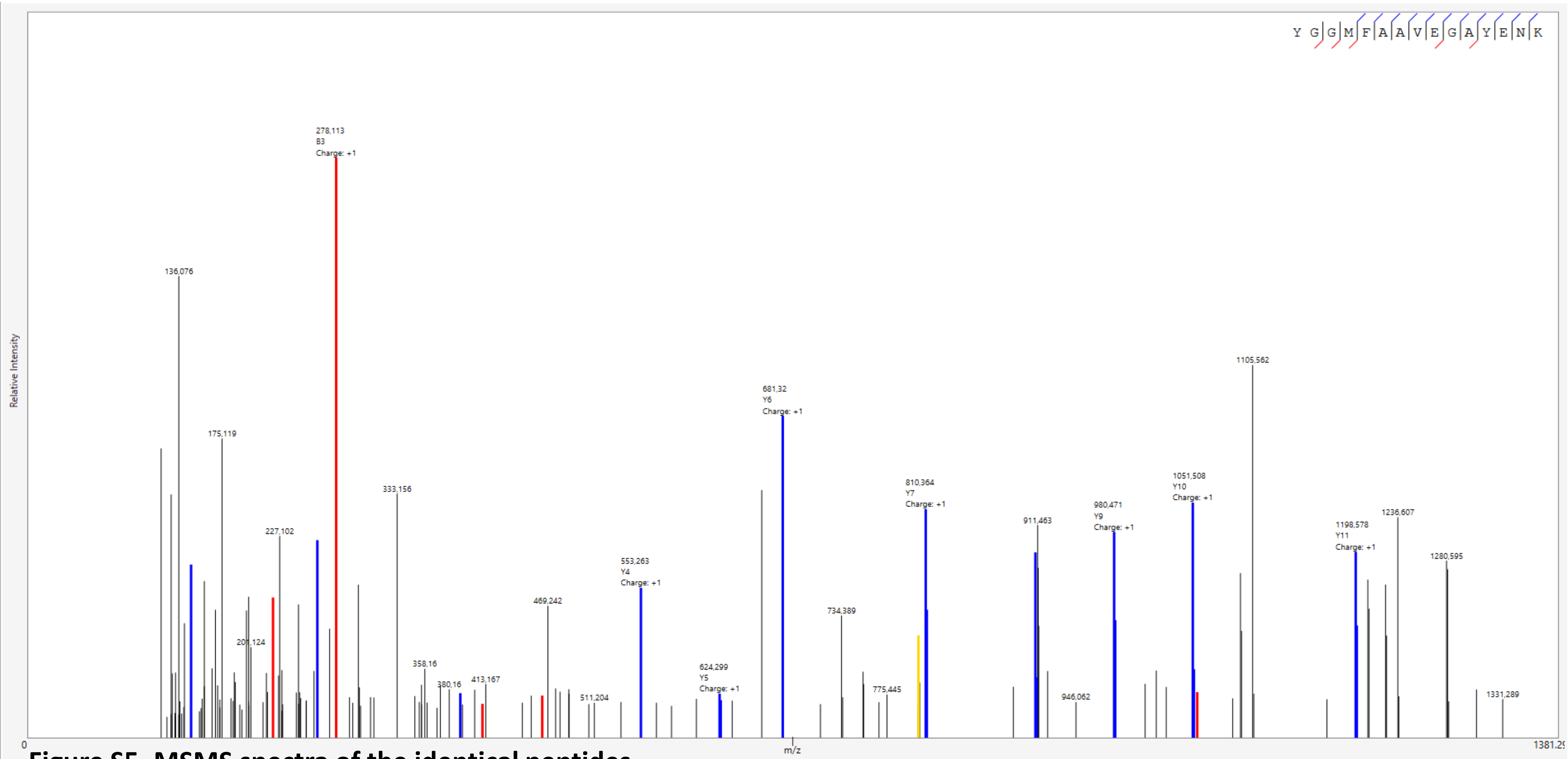

Figure S5. MSMS spectra of the identical peptides.

Mouse Corpus Callosum

ASSHSTDLMEAMAMGSVEASYK

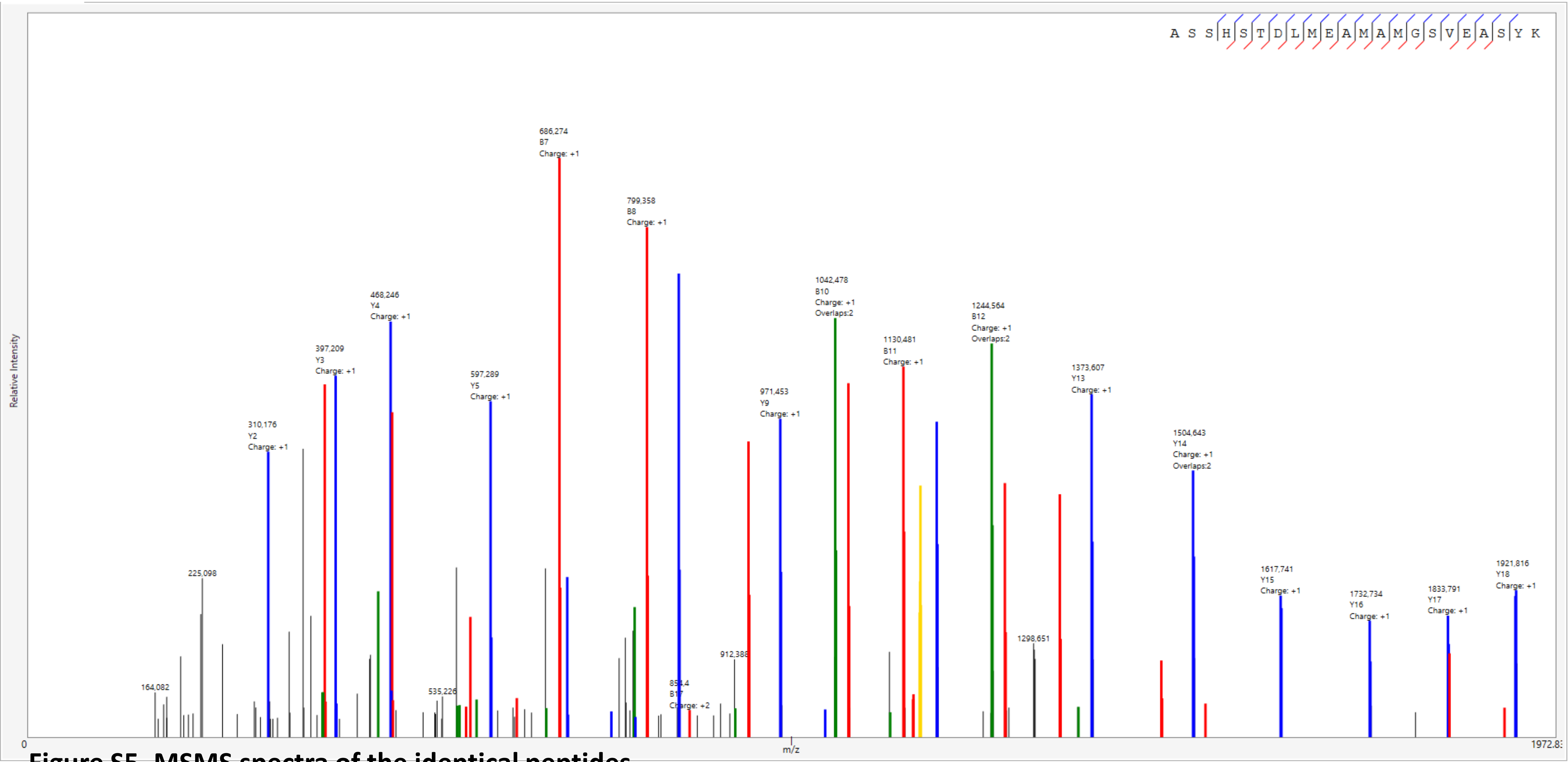

Figure S5. MSMS spectra of the identical peptides.

Mouse Corpus Callosum
CLAAALIVLTESGR

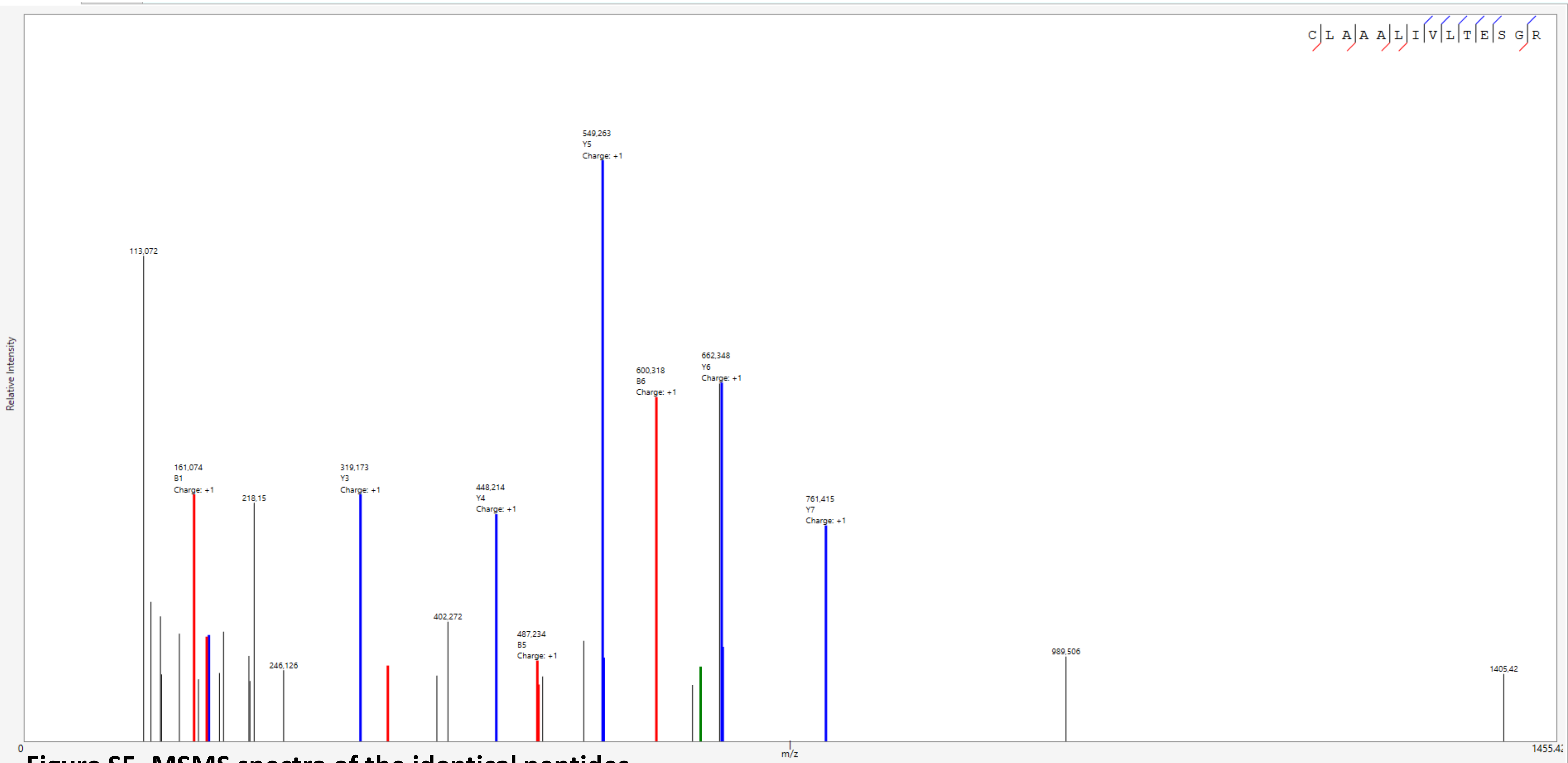

Figure S5. MSMS spectra of the identical peptides.

Mouse Corpus Callosum
EAEAAMFHR

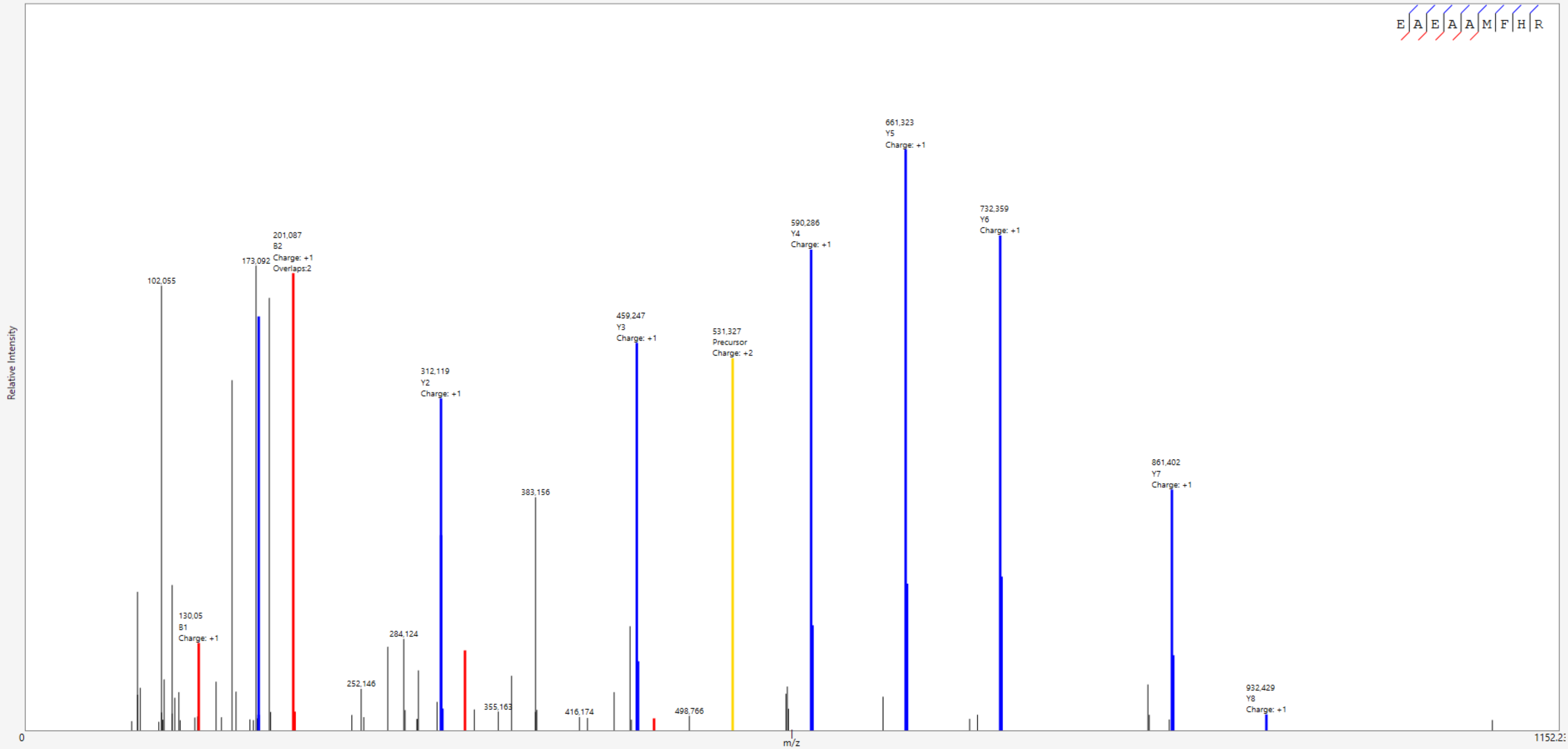

Figure S5. MSMS spectra of the identical peptides.

Mouse Corpus Callosum

IIPTLEEYQHYK

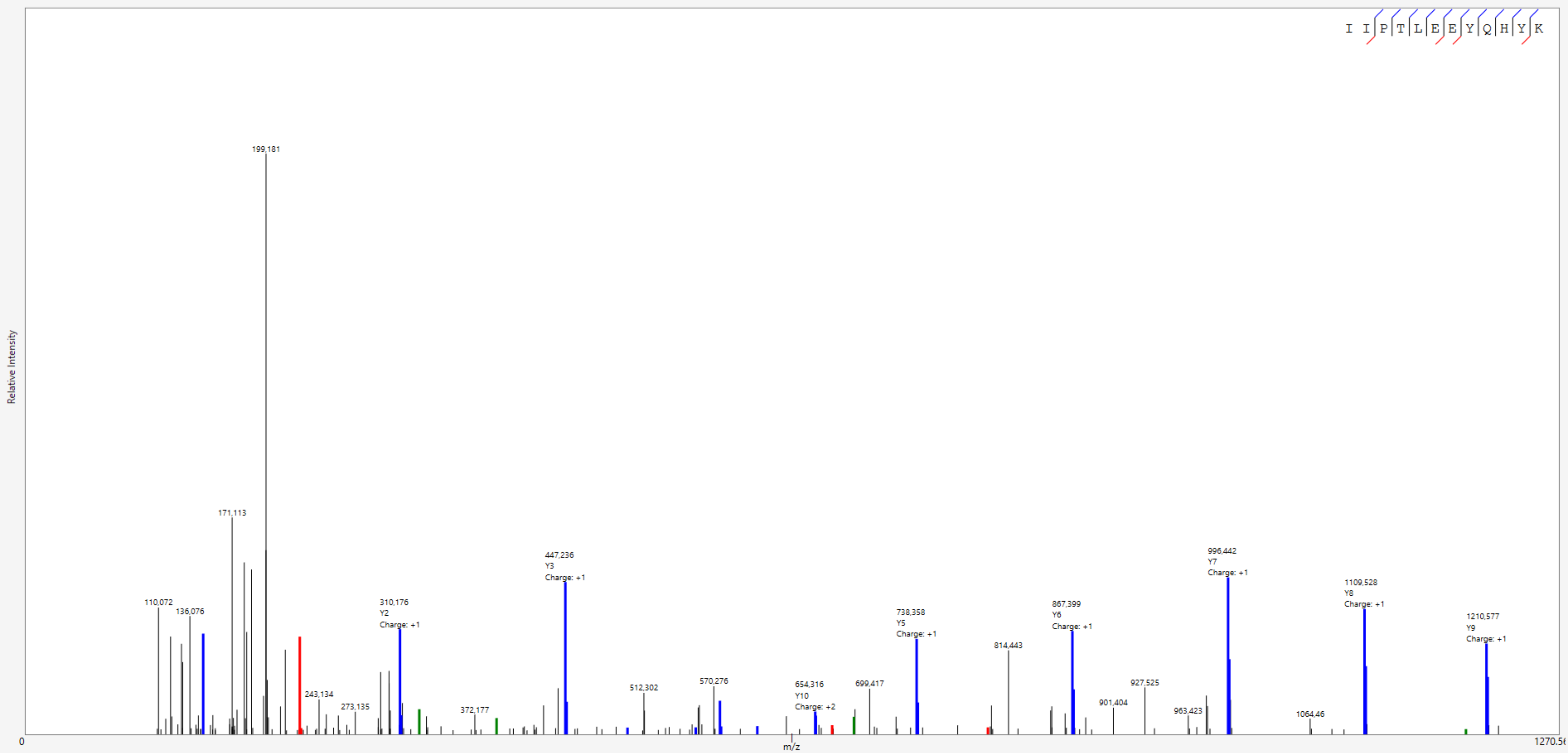

Figure S5. MSMS spectra of the identical peptides.

Mouse Corpus Callosum
LFEELVR

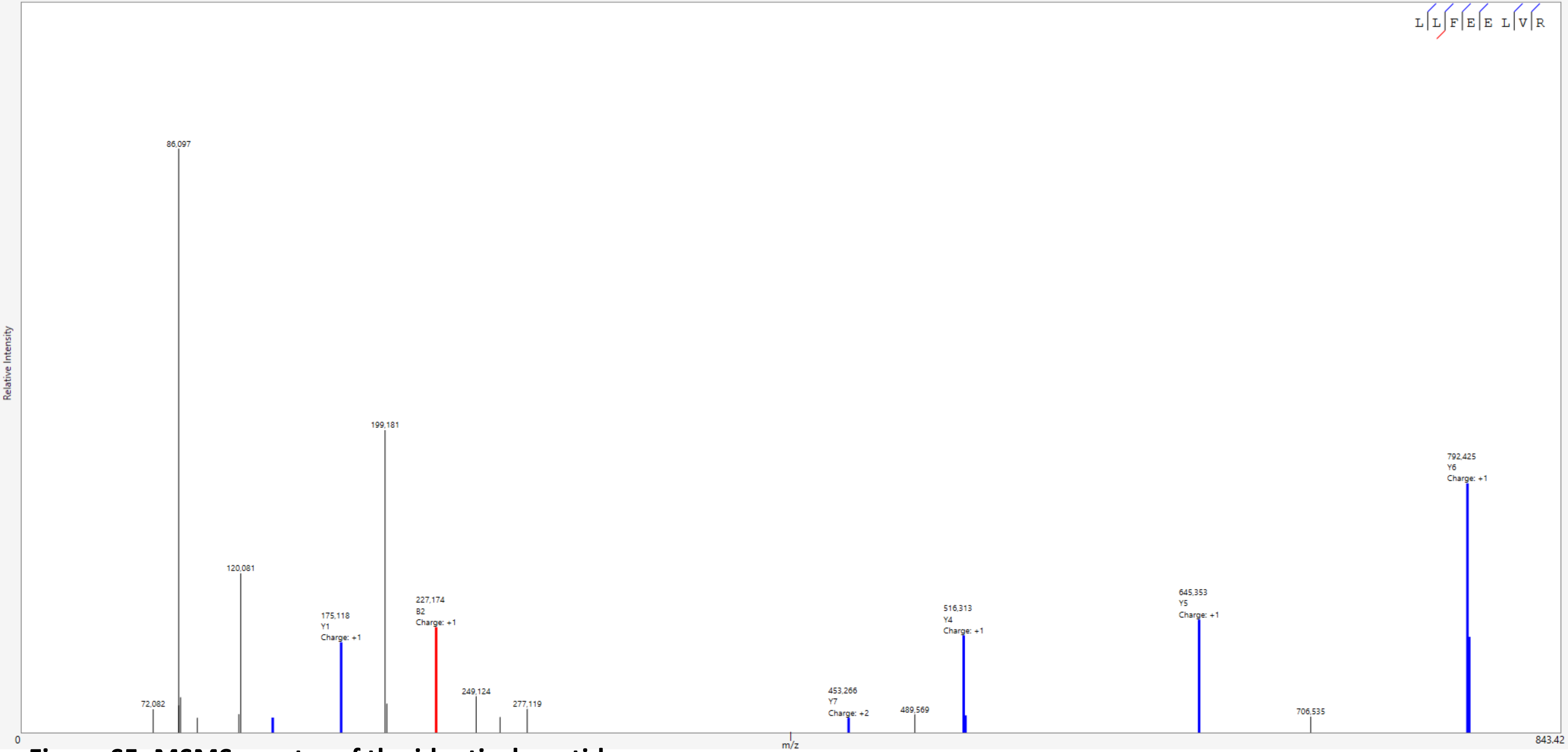

Figure S5. MSMS spectra of the identical peptides.

Mouse Corpus Callosum
NIDQSEFEGFSFVNSEFLKPEVK

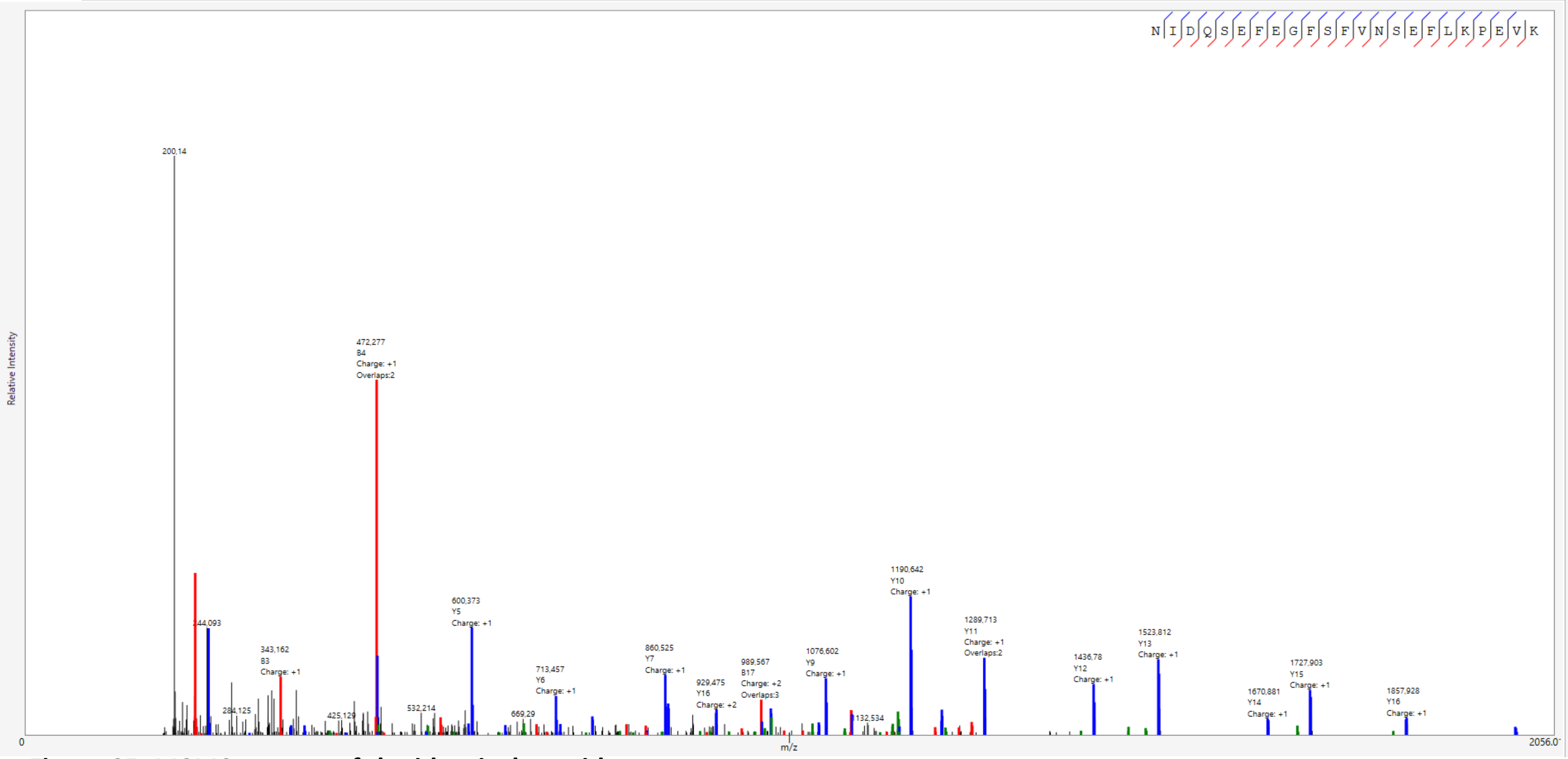

Figure S5. MSMS spectra of the identical peptides.

Mouse Corpus Callosum
YGGMFAAVEGAYENK

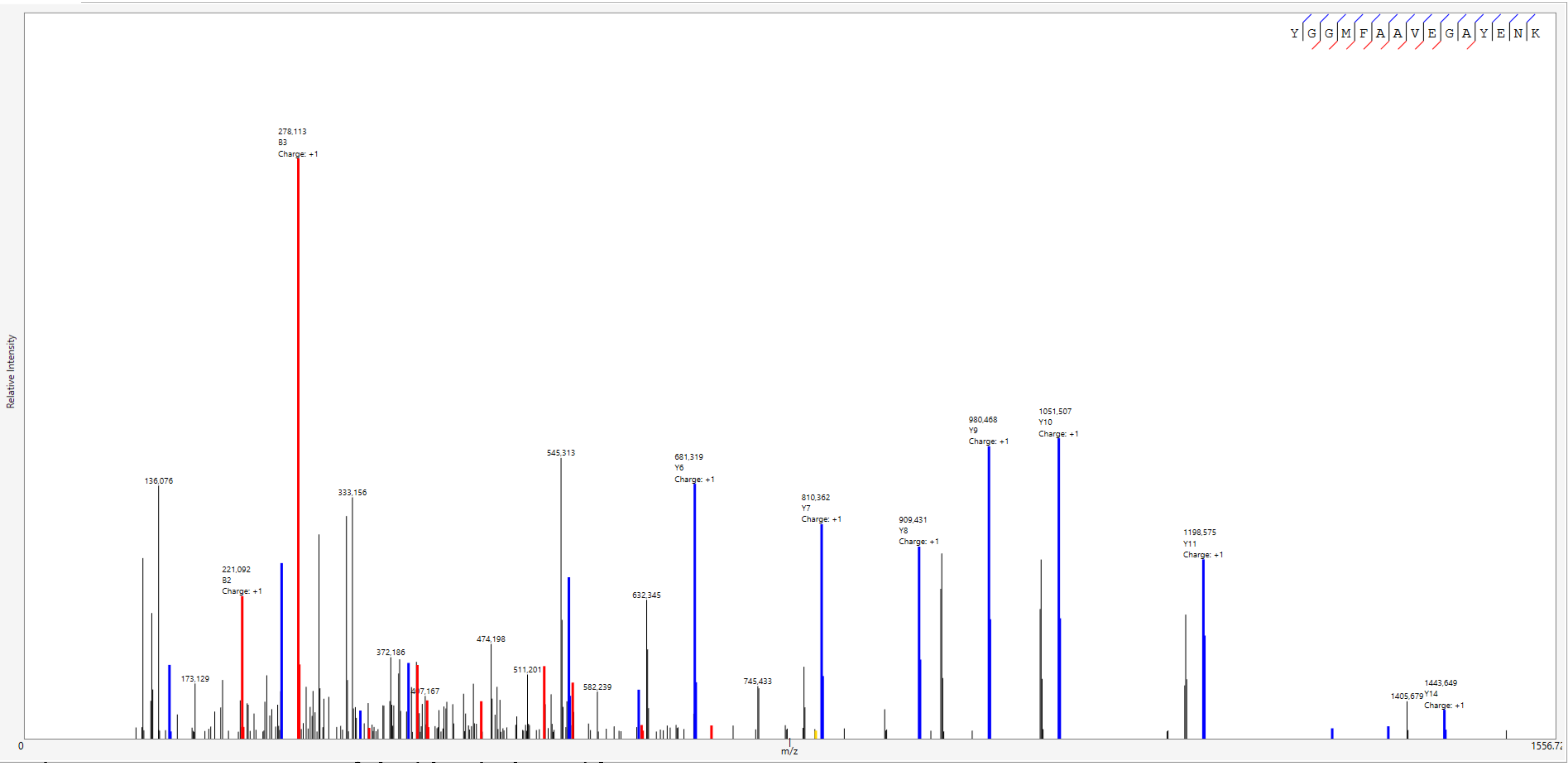

Figure S5. MSMS spectra of the identical peptides.

Mouse Olfactory Bulb

LFEELVR

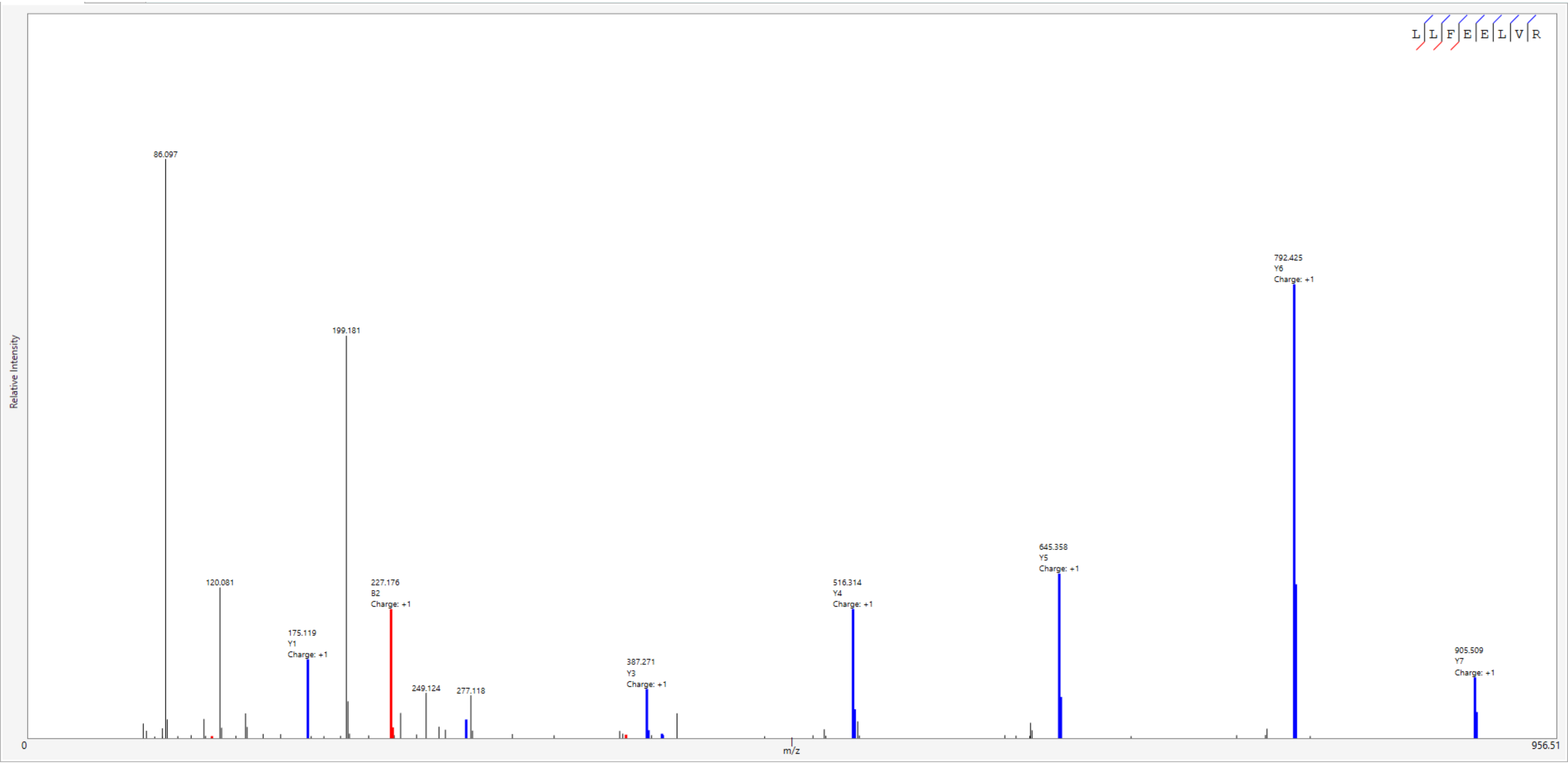

Figure S5. MSMS spectra of the identical peptides.

Mouse Olfactory Bulb
ASSHSTDLMEAMAMGSVEASYK

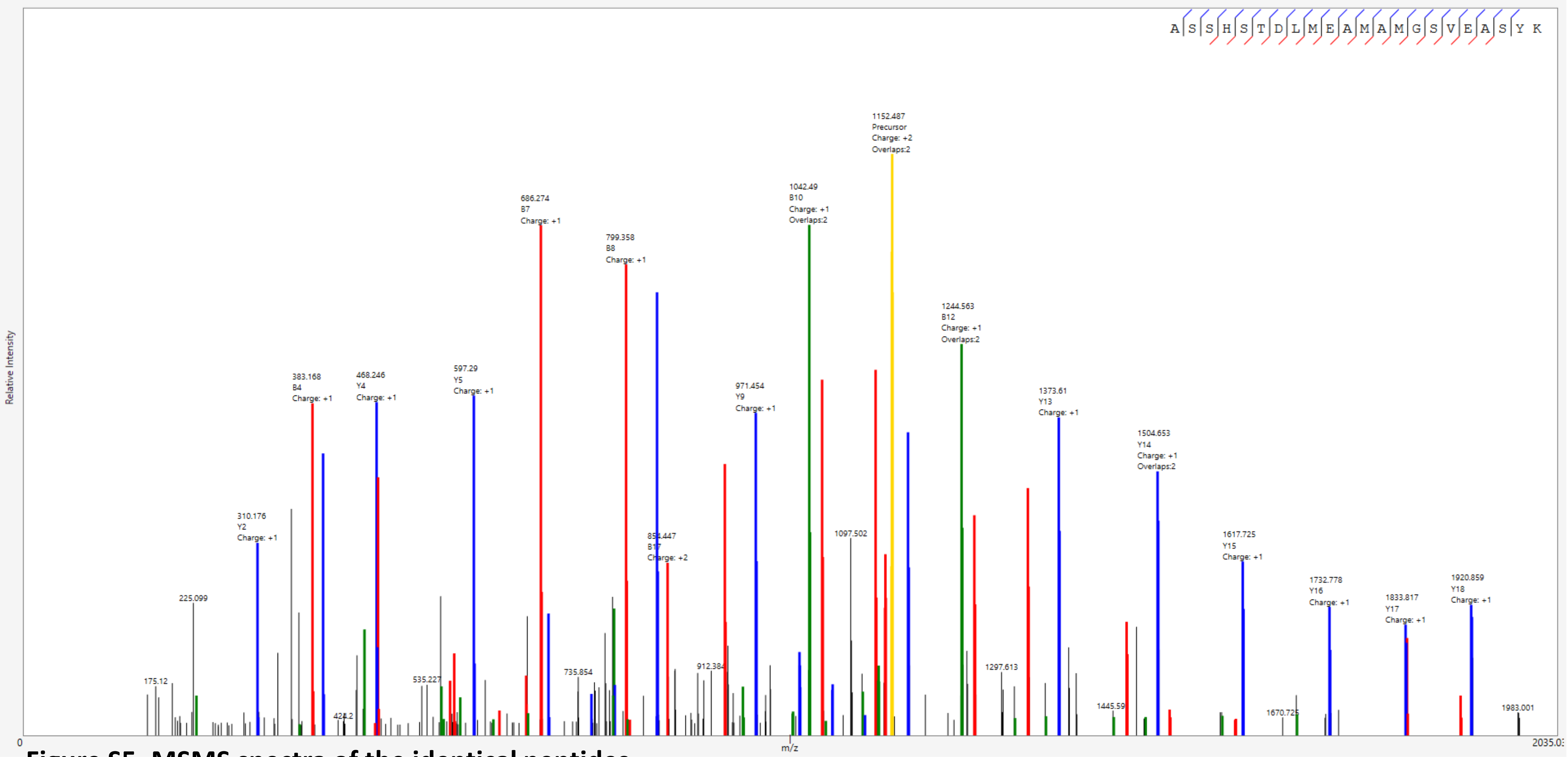

Figure S5. MSMS spectra of the identical peptides.

Mouse Olfactory Bulb
ASSHSTDLMEAMAMGSVEASYK

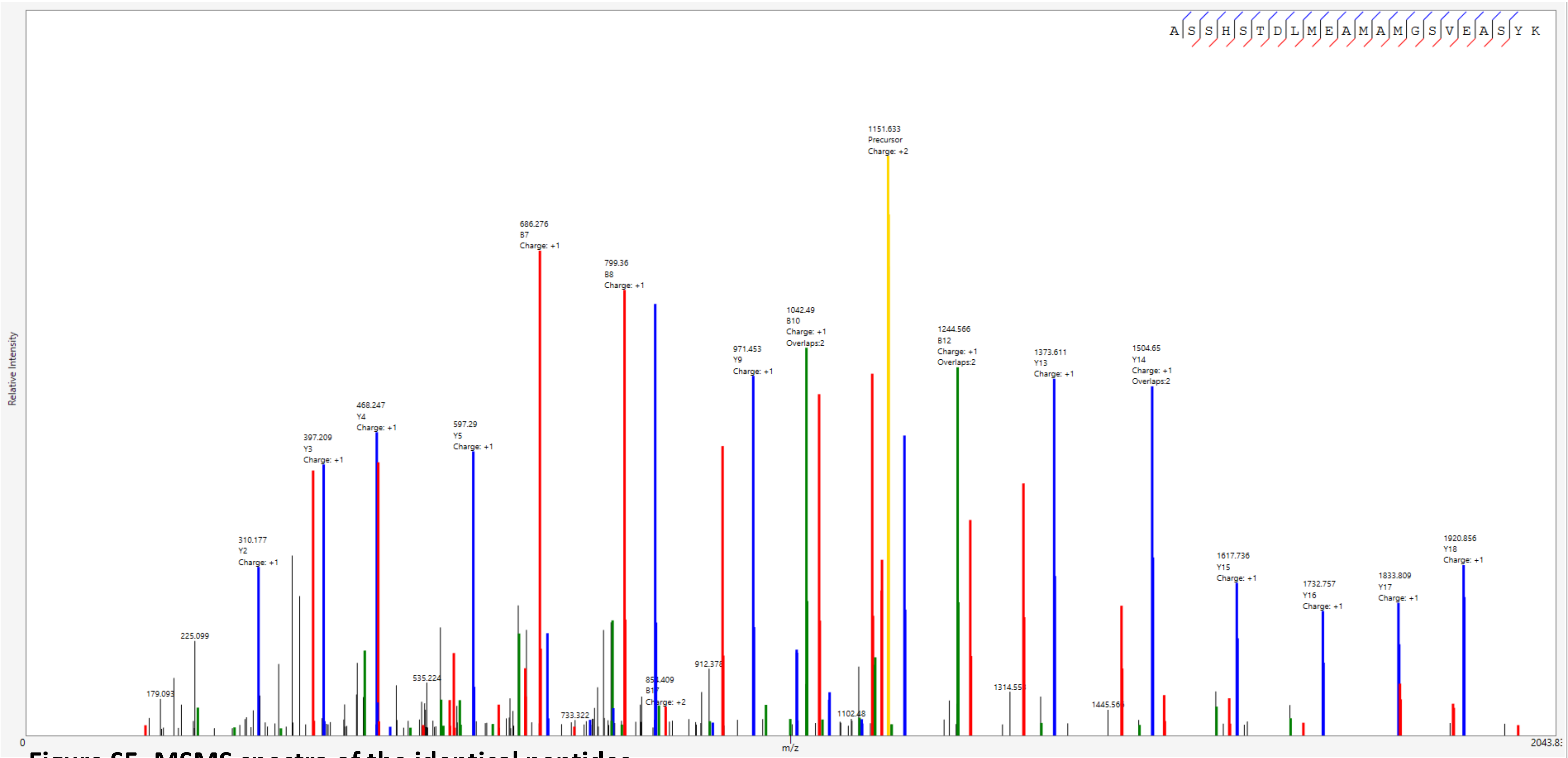

Figure S5. MSMS spectra of the identical peptides.

Mouse Olfactory Bulb
EAEAAMFHR

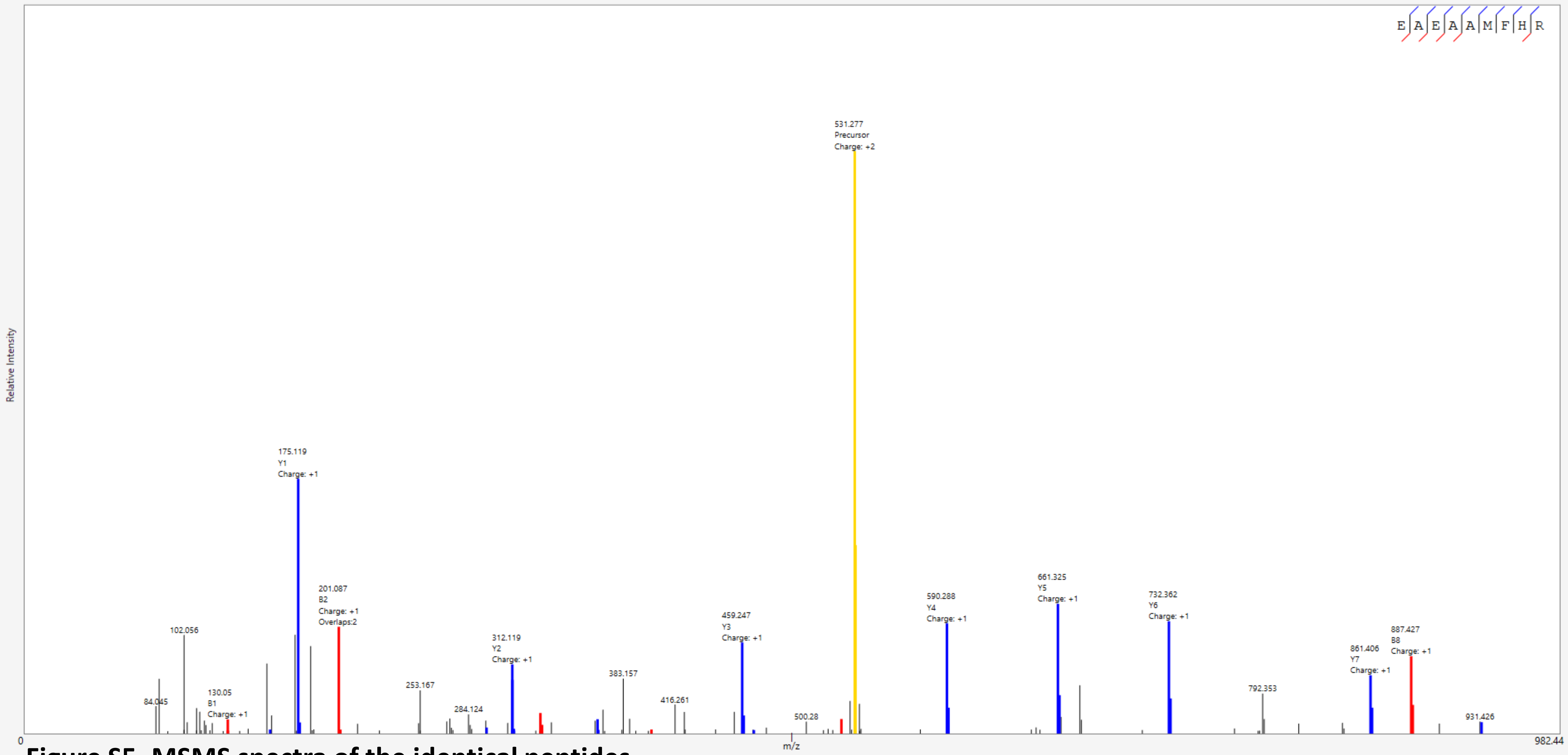

Figure S5. MSMS spectra of the identical peptides.

Mouse Olfactory Bulb
CLAAALIVLTESGR

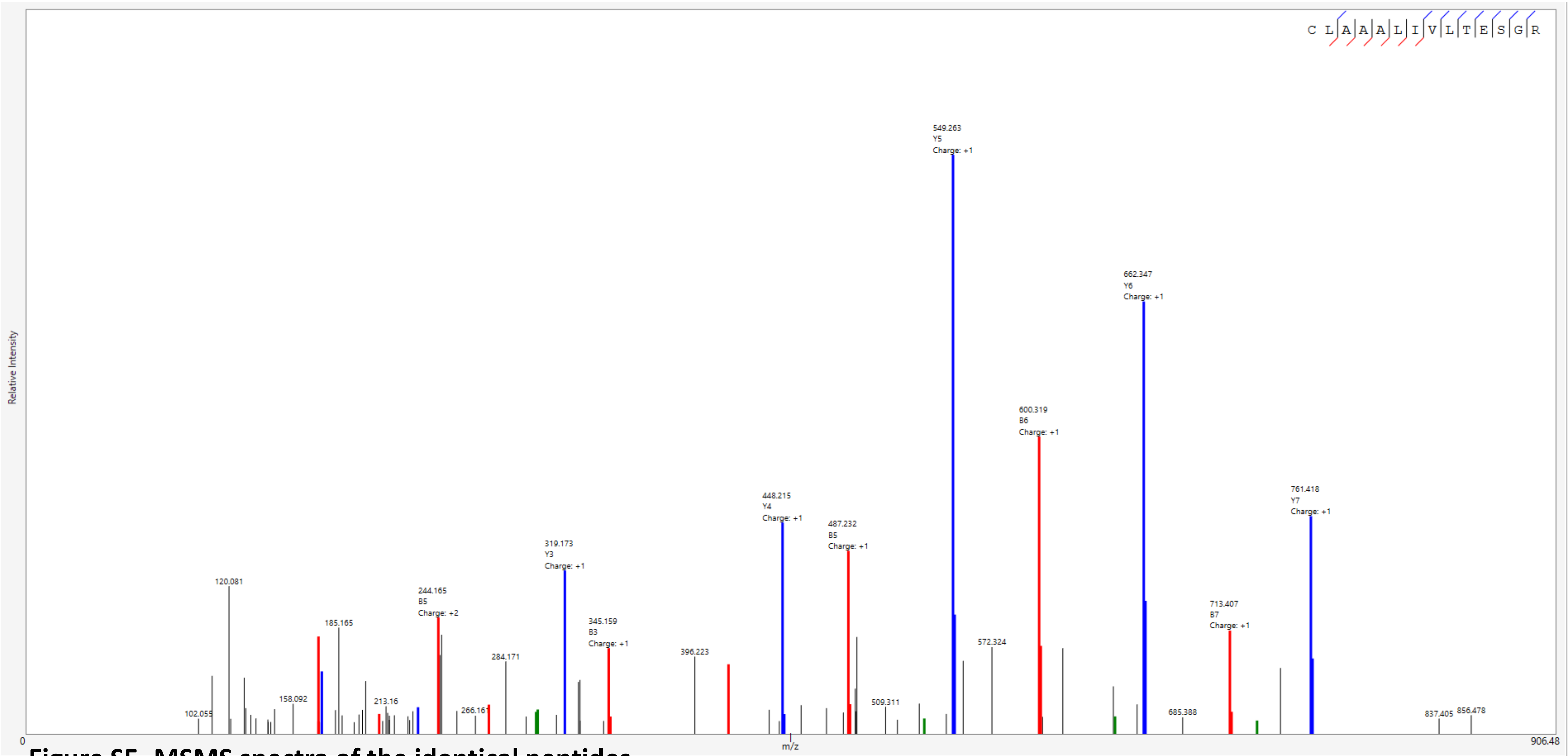

Figure S5. MSMS spectra of the identical peptides.

Mouse Olfactory Bulb
CLAAALIVLTESGR

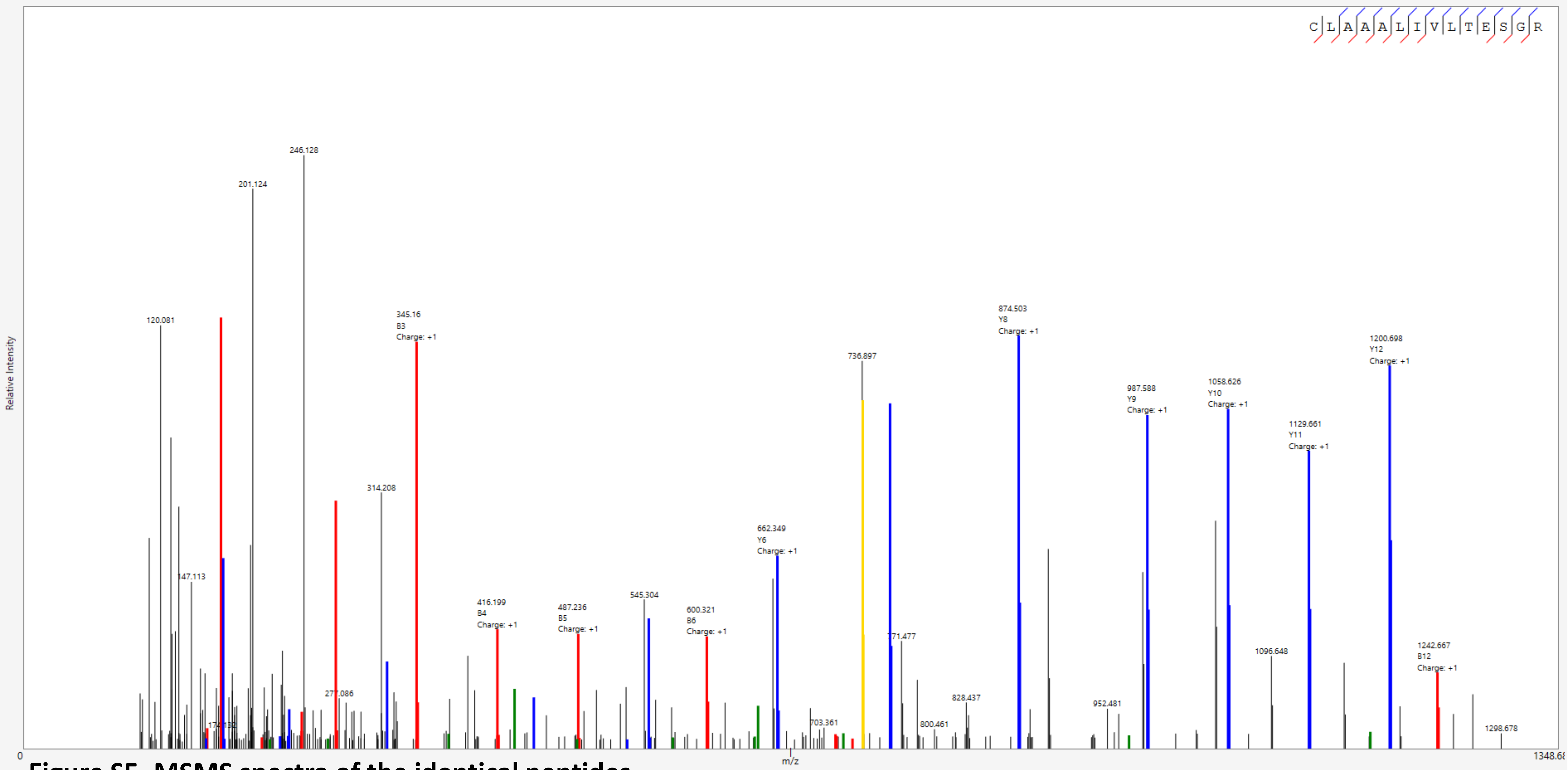

Figure S5. MSMS spectra of the identical peptides.

Mouse Olfactory Bulb
NIDQSEFEGFSFVNSEFLKPEVK

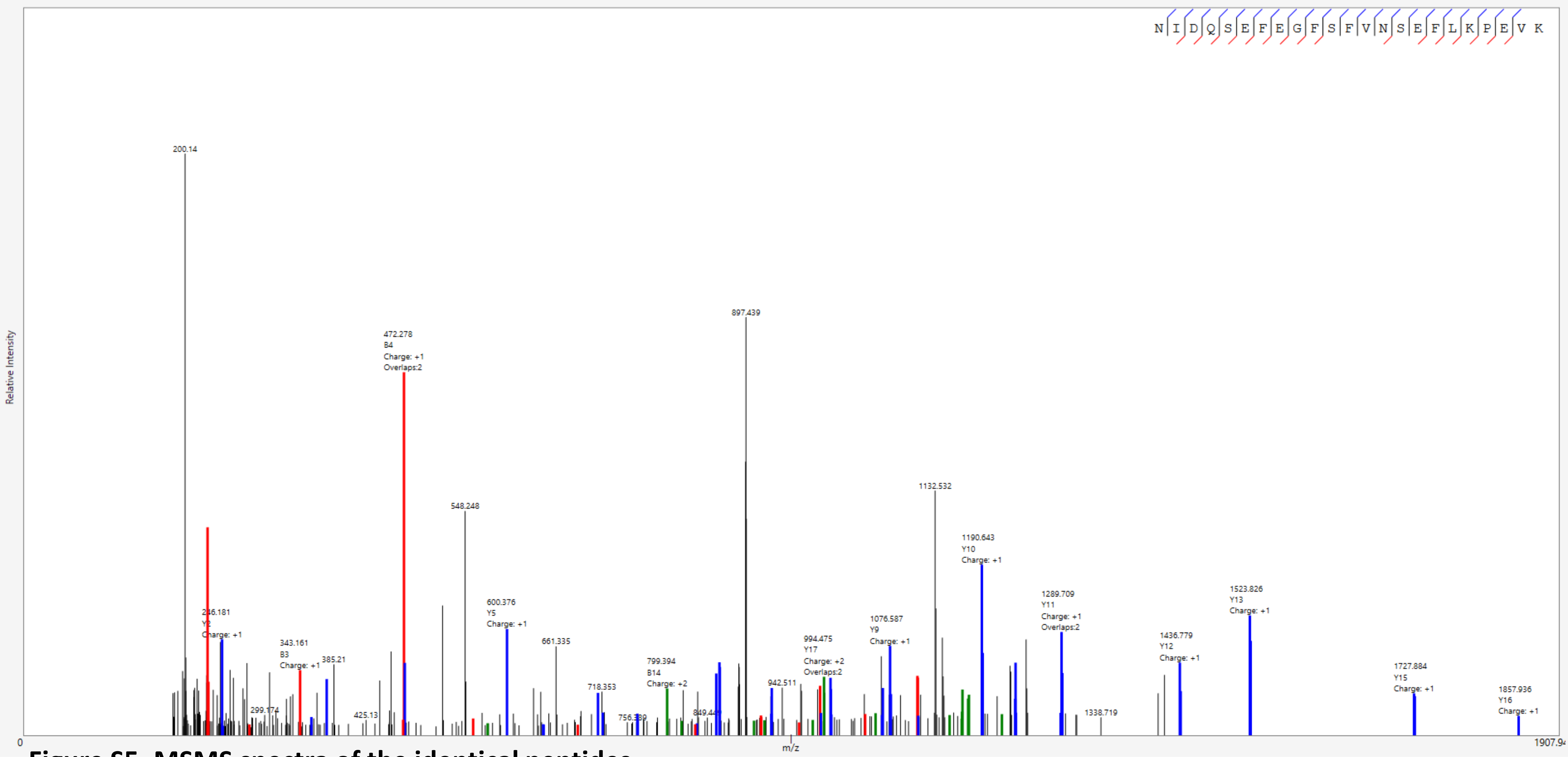

Figure S5. MSMS spectra of the identical peptides.

Mouse Olfactory Bulb
NIDQSEFEGFSFVNSEFLKPEVK

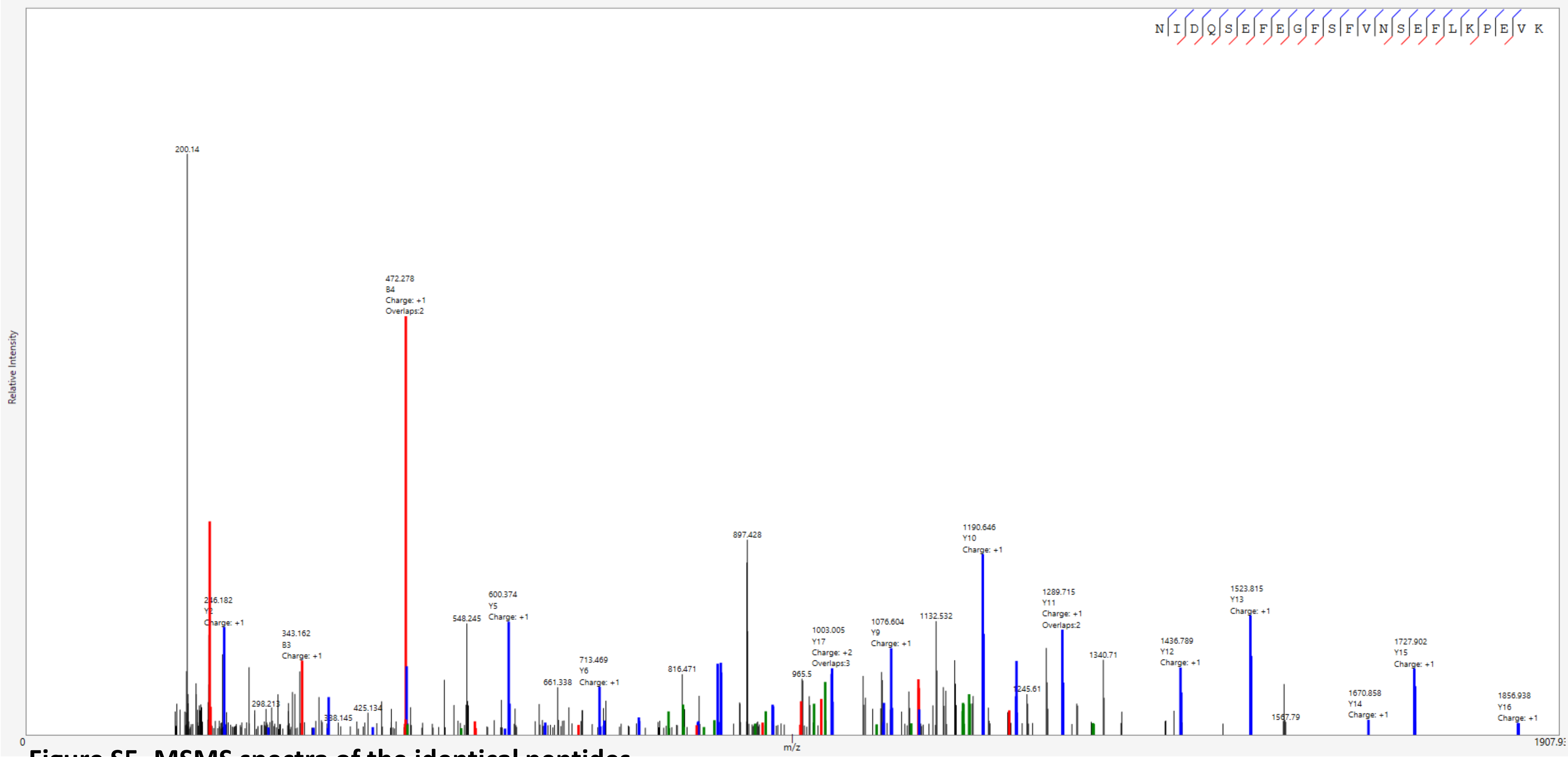

Figure S5. MSMS spectra of the identical peptides.

Mouse Olfactory Bulb
WEVLIGSTHILTPTK

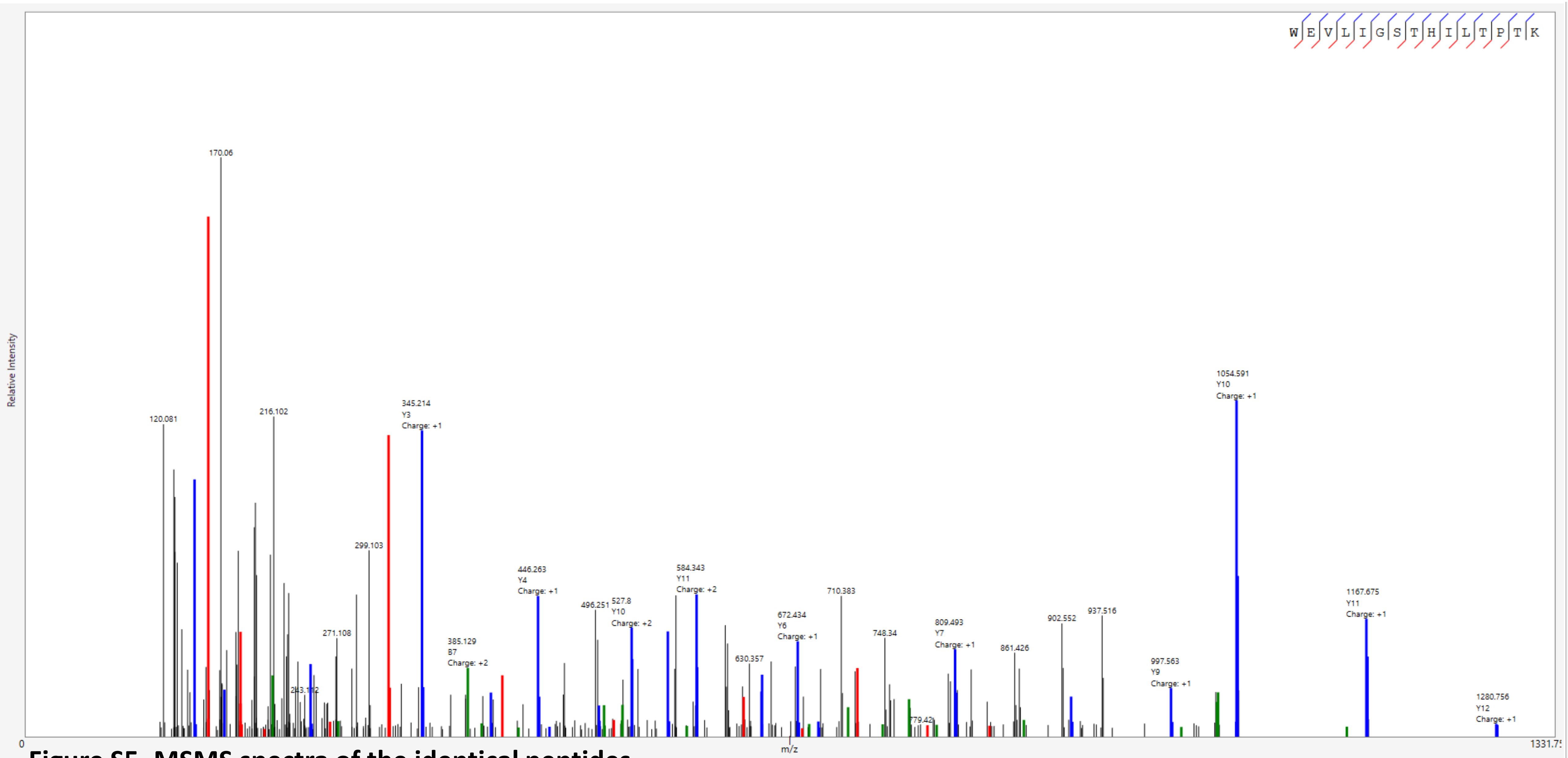

Figure S5. MSMS spectra of the identical peptides.

Mouse Olfactory Bulb
AHSDYMLYVYDSR

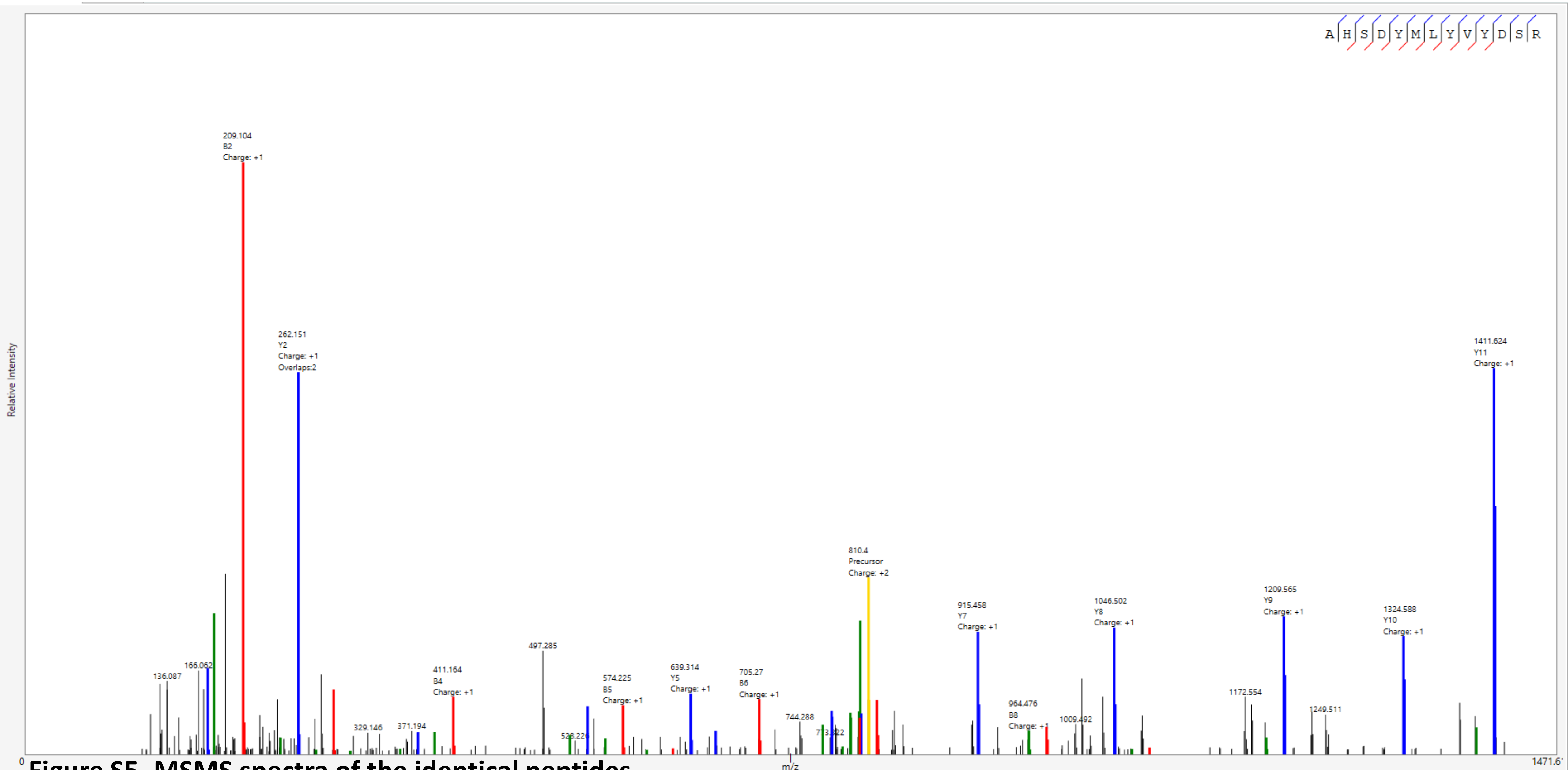

Figure S5. MSMS spectra of the identical peptides.

Human Olfactory Bulb
AHSDYMLYVYDSR

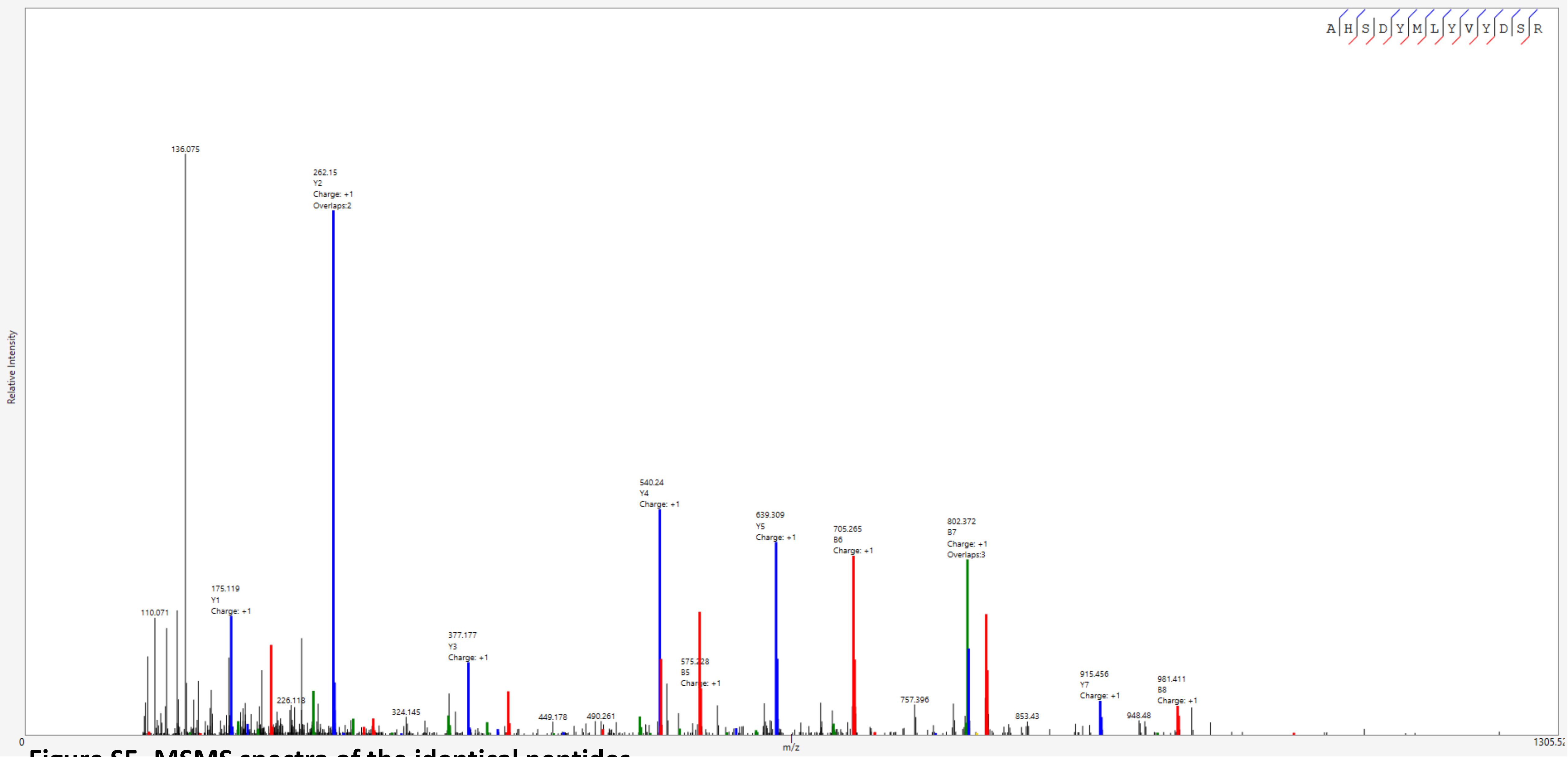

Figure S5. MSMS spectra of the identical peptides.

Mouse Olfactory Bulb

YGGMFAAVEGAYENK

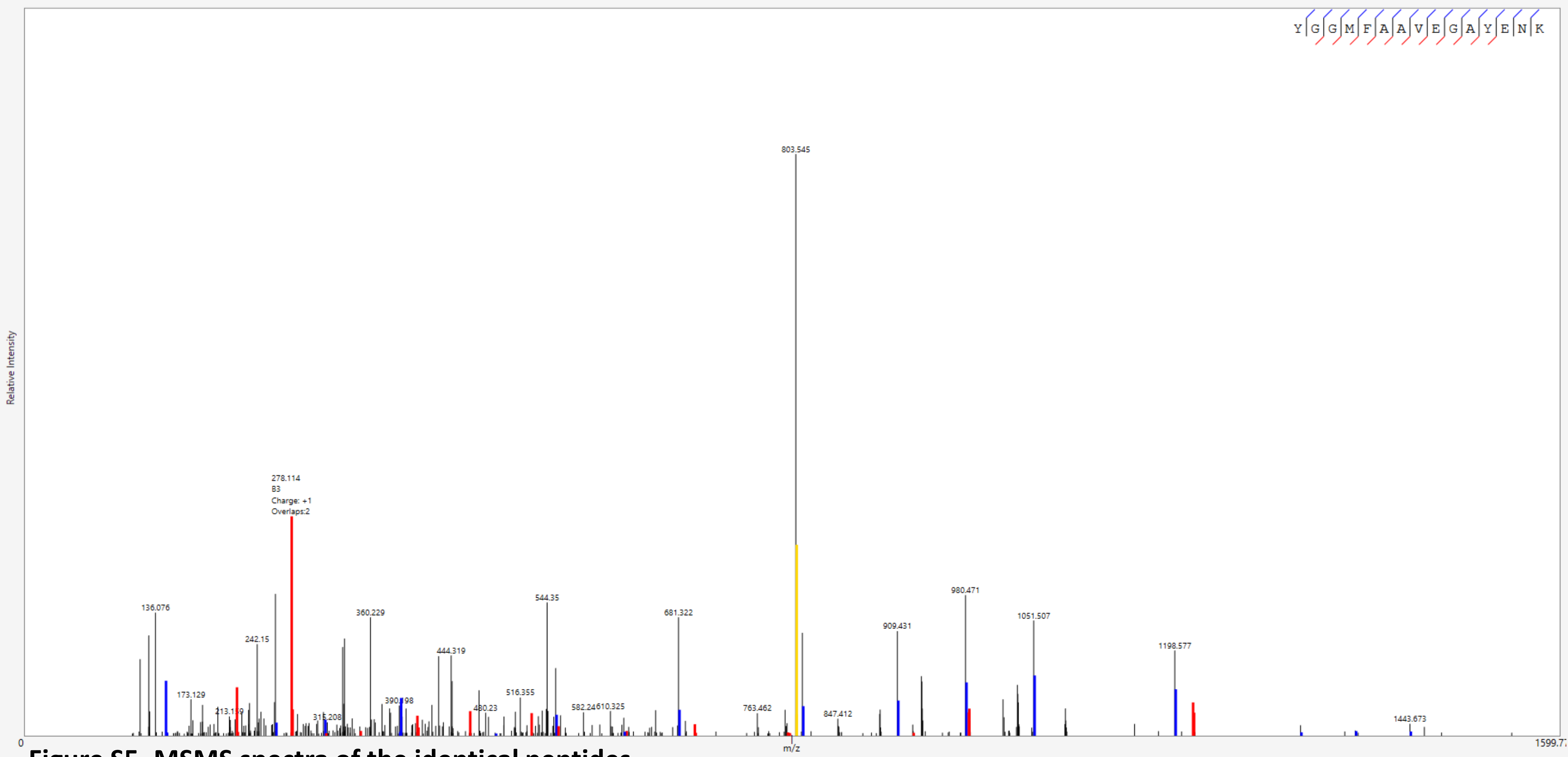

Figure S5. MSMS spectra of the identical peptides.

Mouse Olfactory Bulb
YGGMFAAVEGAYENK

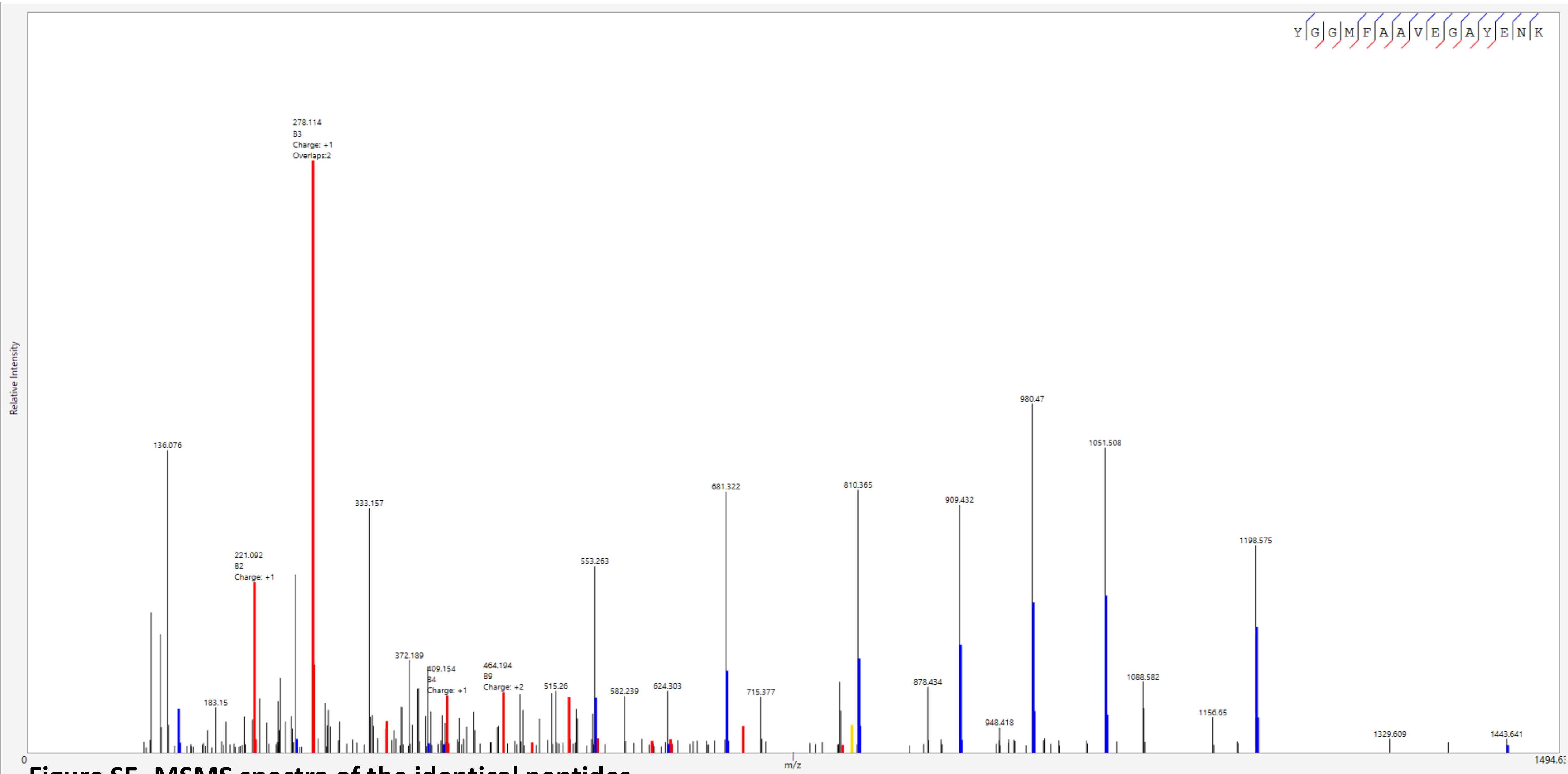

Figure S5. MSMS spectra of the identical peptides.

Mouse Olfactory Bulb
IIPTLEEYQHYK

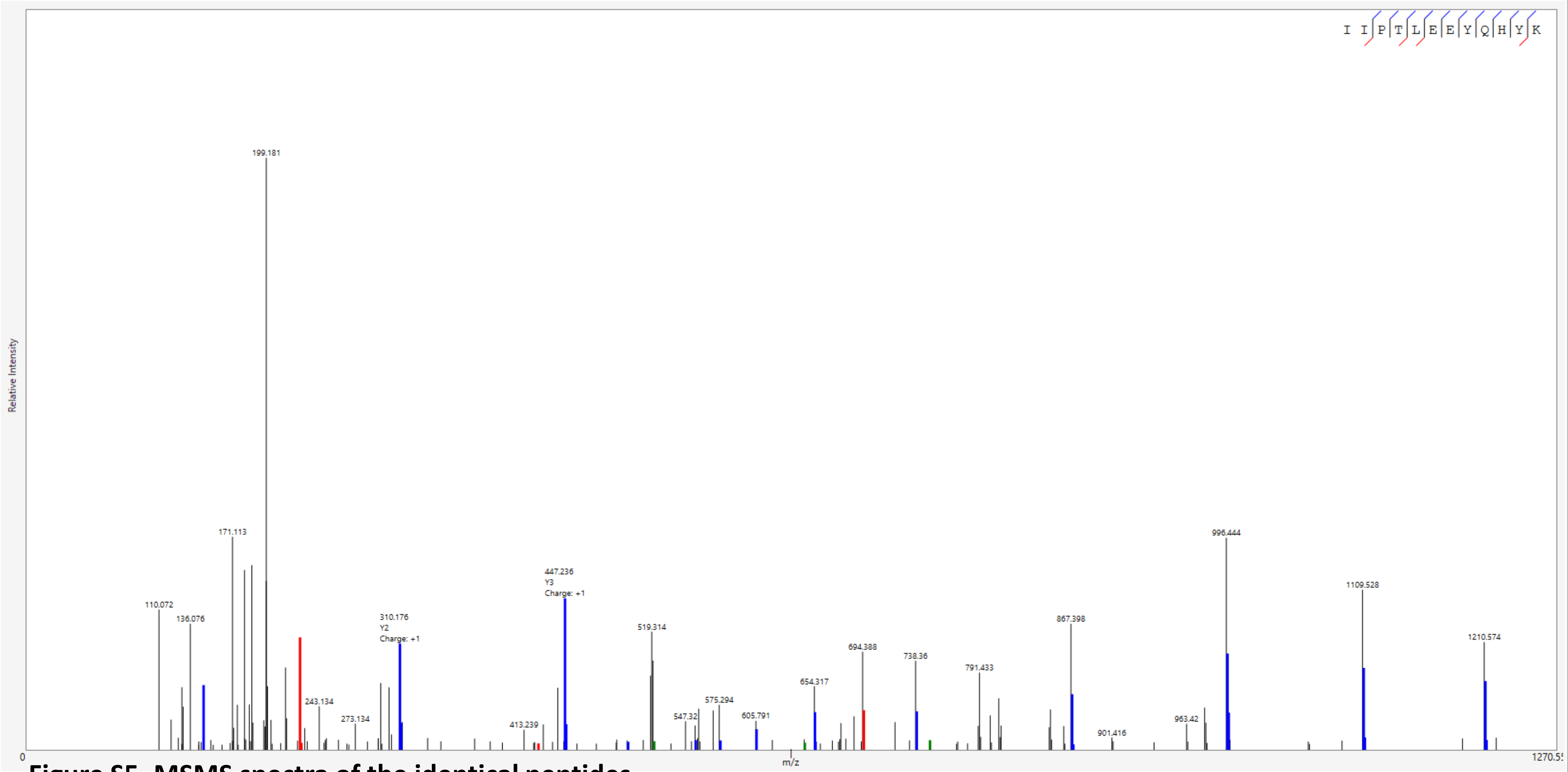

Figure S5. MSMS spectra of the identical peptides.

Mouse Olfactory Bulb
IIPTLEEYQHYK

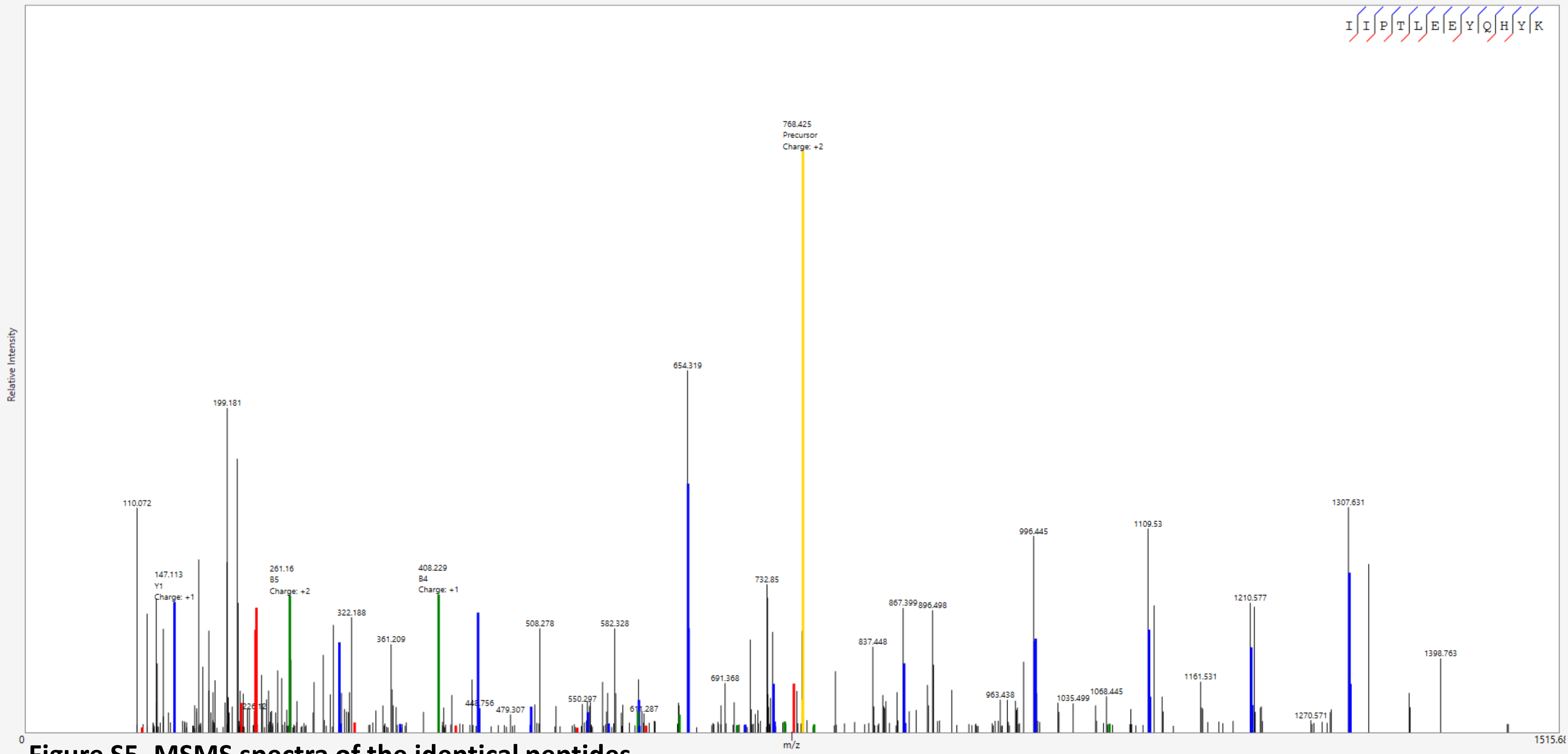

Figure S5. MSMS spectra of the identical peptides.
